# Plant neighbor detection and allelochemical response are driven by root-secreted signaling chemicals

**DOI:** 10.1038/s41467-018-06429-1

**Published:** 2018-09-24

**Authors:** Chui-Hua Kong, Song-Zhu Zhang, Yong-Hua Li, Zhi-Chao Xia, Xue-Fang Yang, Scott J. Meiners, Peng Wang

**Affiliations:** 1 College of Resources and Environmental Sciences, China Agricultural University, 100193 Beijing, China; 2 Department of Biological Sciences, Eastern Illinois University, Charleston, IL 61920 USA; 3 Institute of Applied Ecology, Chinese Academy of Sciences, 110016 Shenyang, China

## Abstract

Plant neighbor detection and response strategies are important mediators of interactions among species. Despite increasing knowledge of neighbor detection and response involving plant volatiles, less is known about how soil-borne signaling chemicals may act belowground in plant–plant interactions. Here, we experimentally demonstrate neighbor detection and allelopathic responses between wheat and 100 other plant species via belowground signaling. Wheat can detect both conspecific and heterospecific neighbors and responds by increasing allelochemical production. Furthermore, we show that (-)-loliolide and jasmonic acid are present in root exudates from a diverse range of species and are able to trigger allelochemical production in wheat. These findings suggest that root-secreted (-)-loliolide and jasmonic acid are involved in plant neighbor detection and allelochemical response and may be widespread mediators of belowground plant-plant interactions.

## Abstract (web summary)

Plant growth and physiology respond to the presence of neighboring plants. Here the authors show that the chemicals (-)-loliolide and jasmonic acid, which were present in the root exudates of a range of plant species, can be detected by wheat and induce allelochemical production.

## Introduction

Plants are capable of detecting and responding to neighboring plants, generating consequences for plant performance and playing important roles in plant coexistence and community assembly^1–3^. Plant neighbor detection involves both physical and chemical signals, including far-red light reflection, alteration of nutrient availability and plant-released secondary metabolites. These signals trigger complex plant response strategies such as shade avoidance, root foraging, and chemical defense^4–6^. Much of the research into chemical-mediated neighbor detection and signaling interactions has dealt with volatiles induced by herbivory or other environmental stressors in initiating defensive responses^7–9^. This focus is primarily driven by the accessibility of aerial tissues and availability of reliable techniques to detect and identify volatile chemicals. Nevertheless, plant neighbor detection and signaling interactions take place both aboveground and belowground. Aboveground signaling interactions are well established and mediated by air-borne chemicals including methyl jasmonate, salicylate, benzoate, and indole, as well as ethylene and several volatile terpenes^10–13^. However, the identity of soil-borne chemicals involved in belowground signaling interactions is largely unknown.

In contrast to aboveground signaling chemicals which move freely in air, the transduction of belowground chemicals requires root-soil interactions. A great deal of recent attention has been paid to belowground signaling interactions among plant species at the root level^14–16^. In particular, root detection and placement patterns may be mediated through root-secreted chemicals^17–20^. These belowground signals are potentially conducted by physical contact of root hairs and tips or conveyed through associated mycorrhizal hyphae^21,22^. However, it is extremely difficult to unambiguously isolate chemical-mediated belowground signaling interactions due to the complexity of plant-soil interactions. Specifically, it has been difficult to identify root-secreted signaling chemicals and determine whether their delivery is dependent on root contact or common mycorrhizal networks.

When neighboring plants are detected, focal plants may respond to neighbors in morphological and biochemical ways^23,24^. In particular, neighbors influence plant defensive biochemistry in a species-specific fashion, altering the production of defensive metabolites such as allelochemicals^24,25^. Allelochemicals can have profound effects on the performance of neighboring plants, i.e., allelopathy, an interference mechanism in which neighbor plants are chemically suppressed through the release of allelochemicals from focal plants^26^. However, allelopathy research has primarily focused only on focal plants and their allelochemicals rather than on the signaling interactions between plants that mediate the interaction^27–29^. Therefore, allelopathic interference and neighbor detection usually are studied separately, despite their necessary linkage in nature.

For two species that coexist, plants first may detect and potentially recognize their neighbors, and then initiate allelopathic interference to regulate inter-specific or intra-specific interactions. Neighbor detection and allelochemical response are two inseparable processes when one or more plants occur together and interact^30^. This pattern may arise through the production and release of signaling chemicals that induce the production of defensive allelochemicals. Accordingly, the signaling chemicals may ideally be common to most potential competitors. Alternatively, allelochemical production may be constitutive, produced whether or not neighbors are present, potentially wasting valuable plant resources^26^.

In previous studies we used production of the putative allelochemical DIMBOA (2,4-dihydroxy-7-methoxy-1,4-benzoxazin-3-one), which has a charcterized metabolic profil in soil^31–34^, by allelopathic wheat (*Triticum aestivum*) as a model system. We reported that production of DIMBOA was induced by a diverse range of neighboring plants in a density-dependent manner^29,30^. Root exudates from a range of species were able to induce DIMBOA production and the plant hormone jasmonate, and to a lesser extent salicylate, were able to induce DIMBOA production^30^. Despite these findings, the identity of the soil-borne chemical signals that are responsible for neighbor detection and allelochemical response in belowground signaling interactions has not been exhaustively characterized and whether such soil-borne chemical signals are common to most plant species remains unknown.

Here, we show that, using new data and data from previous studies we show that wheat can respond to at least 100 other plant species via belowground interactions. We show that (-)-loliolide and jasmonic acid, present in root exudates are sufficient to induce this response and suggesting that they can mediate this response.

## Results

### Neighbor-induced allelochemical responses

**Fig. 1:**
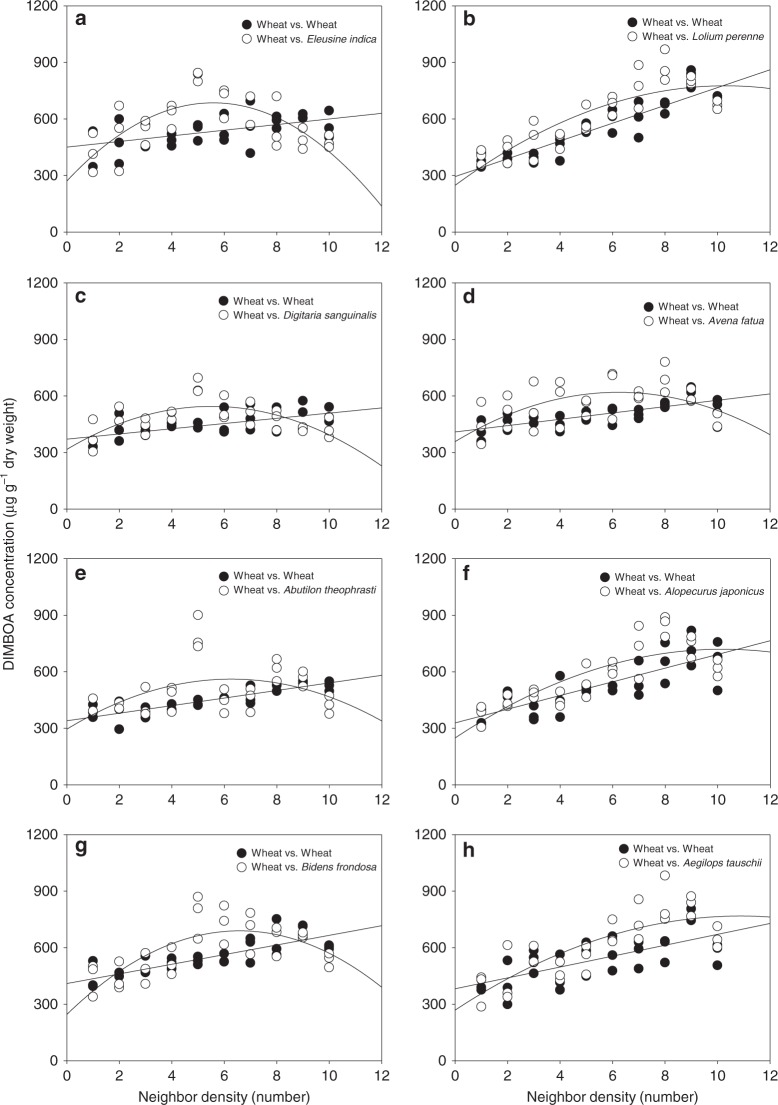
Variation in allelochemical DIMBOA concentration of wheat plants in response to eight commonly interacting weeds at different densities. **a** *Eleusine indica*; **b** *Lolium perenne*; **c** *Digitaria sanguinalis*; **d** *Avena fatua*; **e** *Abutilon theophrasti*; **f** *Alopecurus japonicus*; **g** *Bidens frondosa*; **h** *Aegilops tauschii*. Regression lines were fitted as the model results of the second-order polynomial of neighbour density and its interaction with species identity (wheat-weed), or a linear model (wheat-wheat). Significance of polynomial terms, e.g., neighbor density (*D*), density-squared (*D*^2^), species (*S*), *D* × *S*, and *D*^2^ × *S* are shown in Supplementary Table 1

**Fig. 2:**
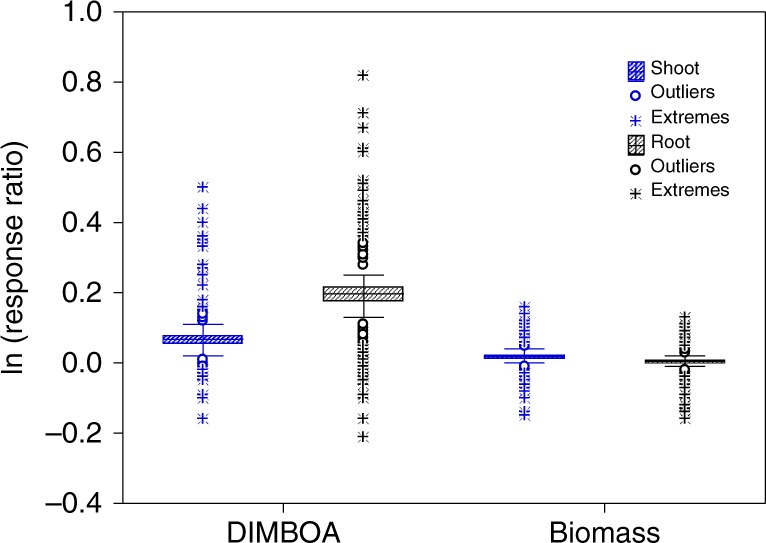
Biomass and allelochemical response of wheat to the presence of 100 heterospecific neighbors at a 5:5 mixture. Change in biomass or the allelochemical DIMBOA was calculated for each species by ln (T/C), based on the wheat dry weight or allelochemical quantities in wheat-neighbor treatments (T) and in wheat–wheat controls (C). Values equal to controls are represented by 0, greater values indicate increased growth or DIMBOA relative to controls. Box whiskers extend to 1 standard error around the means

Previously we reported a density dependent increase in DIMBOA concentration in wheat roots when co-cultivated with multiple weed species results^29,30^. We investigated density-dependence of the DIMBOA response further by co-cultivating wheat with eight weed species (four of which were examined previously^30^). When wheat was paired with itself and eight common weeds (*Eleusine indica, Digitaria sanguinalis, Abutilon theophrasti*, *Bidens frondosa, Lolium perenne, Avena fatua, Alopecurus japonicus*, and *Aegilops tauschii*) that often come into contact with wheat, allelochemical DIMBOA concentration varied with the density of the neighbors. Significantly increased concentrations of the allelochemical were observed at the 5:4–5:9 (wheat: neighbor) proportions (Fig. 1; Supplementary Table 1). It appeared that the presence of either conspecific or heterospecifc neighbors could induce the allelochemical response in a density-dependent manner. To provide a broader context for the neighbor-induced allelochemical response, we combined data from our previous publication where DIMBOA concentration was examined in wheat co-cultivated with 36 different wheat species^29^ with newly generated data for additional species. This produced a dataset of wheat biomass and wheat DIMBOA concentration in root and shoot following co-cultivation with a total of 100 different plant species at ratios of 5:8 and/or 5:5 (Supplementary Table 2). We found markedly increased induction of DIMBOA in heterospecific combinations, relative to DIMBOA production when competing with conspecifics, particularly in wheat roots (Fig. 2). Significant increases in wheat DIMBOA production occurred in 38/100 species at a 5:5 proportion while 70/100 species at a 5:8 mixture (Supplementary Table 2). This effect was much larger in wheat roots than in shoots, with an effect size in roots nearly three-fold larger than that of shoots. In sharp contrast to the neighbor-induced allelochemical response, the presence of heterospecific neighbors led to very little changes in wheat biomass of shoots and roots relative to controls (Fig. 2). Though statistically significant, increases in shoot biomass relative to those in conspecific controls were minimal. The timing of species interactions also had a significant impact on DIMBOA concentrations. Compared with the simultaneous planting of wheat and neighbors at a 5:5 proportion, the early presence of neighbors led to a greater increase of DIMBOA concentration, while the later colonization of neighbors did not induce an allelochemical response, being nearly identical to intraspecific controls (Supplementary Fig. 1). Though significant intraspecific induction occurred in wheat, the responsiveness was by far the least sensitive, showing that neighbor-induced allelochemical responses are more related to the density and early exposure to heterospecific neighbors, than the identity of the competing neighbor species.

### Chemical-mediated belowground signaling interactions

**Fig. 3:**
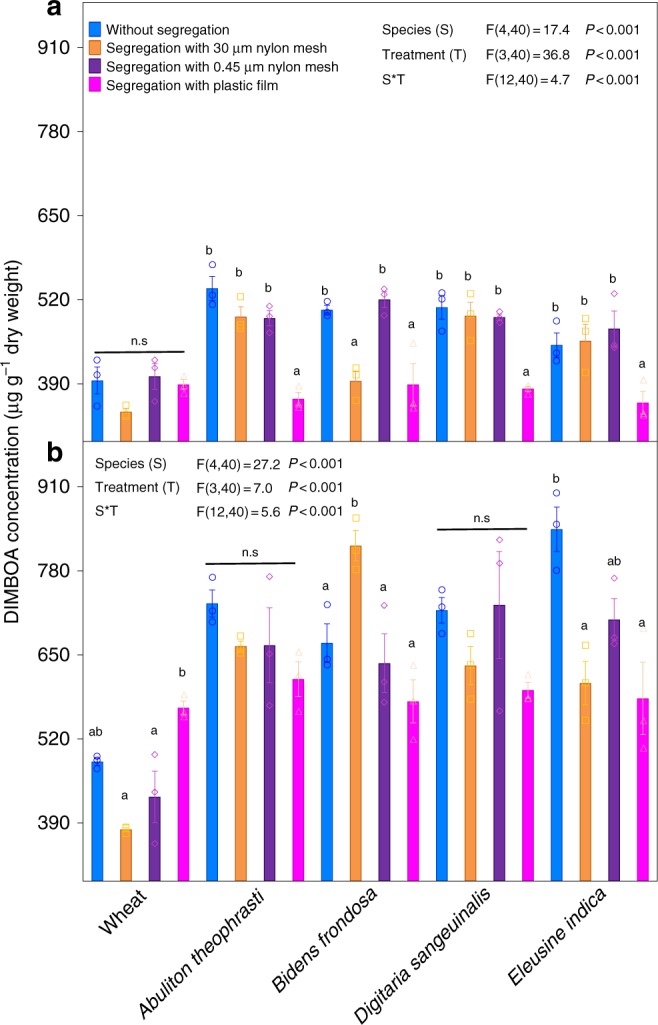
Allelochemical response of wheat shoot and root to the presence of neighbors grown at a 5:5 mixture with various levels of belowground segregation. **a** Wheat shoot; **b** Wheat root. The effect of levels of belowground segregation and species identity on the concentration of allelochemical DIMBOA was tested using linear model with levels of belowground segregation and species as fixed factors. Tukey post hoc tests were calculated between treatments of levels of belowground segregation. Values plotted are means (*n* = 3) ± standard errors (SE). Columns with different letters indicate significant difference among treatments at *P* < 0.05. The error bar denotes 1 SE

Previously we found that DIMBOA concentration of wheat roots was increased even when roots were segregated using 30 μm mesh that prevents physical contact, but not potential chemical or bacterial signals^30^. The mechanisms underlying neighbor detection that generated the allelochemical response were investigated further by belowground segregation experiments using not only 30 μm mesh, but also 0.45 μm mesh and plastic film (Supplementary Fig. 2). The presence of all four heterospecific neighbors (*E. indica, D. sanguinalis, A. theophrasti* and *B. frondosa*) increased DIMBOA concentration in all treatments other than those completely separated with plastic film (Fig. 3). The plastic film completely blocked belowground physical, chemical and biological interactions so that wheat and neighbor interactions were limited to aboveground. The lack of variation in the concentration of DIMBOA with complete exclusion indicated that there were not aerial signaling interactions. However, DIMBOA concentration was increased in the presence of all four neighbors even with segregation by fine nylon mesh, indicating belowground signaling interactions between wheat and neighbors. In particular, the 0.45 μm mesh not only prevented penetration of roots but also blocked mycorrhizal linkages. Under this treatment, only belowground bacterial and chemical interactions occurred in the experimental pots. These results were verified in experiments with four other neighbors (*L. perenne, A. fatua, A. japonicas* and *A. tauschii*). Although these four neighbors did not significantly induce DIMBOA production at a 5:5 mixture, significant induction occurred at a 5:8 mixture, even under belowground segregation with the 0.45 μm mesh (Supplementary Fig. 3; Supplementary Table 3). These results suggest that neighbor-induced allelochemical response was mediated by root-secreted chemical signals or soil bacteria rather than by root contact and soil mycorrhizal hyphae.

### Root-secreted signaling chemicals

To confirm that chemicals and not bacterial interactions were responsible for the observed allelochemical induction, we previously used root exudate extracted from four common weeds and showed that this could induce DIMBOA production^30^. We also previously showed that jasmonic acid (JA), and salicylic acid (SA) when supplied at higher concentration, were sufficient to induce the production of DIMBOA^30^. Here, we extended this analysis by re-analyzing those samples and adding four additional species. The root exudates from neighbor species were applied to the soil at varying concentrations. When wheat was exposed to the root exudates after 6 h, the concentration of allelochemical DIMBOA varied with neighbor species identity in a dose-dependent manner (Supplementary Fig. 4). Induction of DIMBOA was very limited in conspecific exudates when compared to all heterospecific exudates. The root exudates of *A. theophrasti* significantly increased the DIMBOA concentration at a low density of 50 plants 500 mL^−1^, followed by *D. sanguinalis, E. indica*, and *B. frondosa. A. fatua* and *A. tauschii* at a medium concentration of 100 plants 500 mL^−1^. All root exudates from either wheat itself or other species tested significantly induced the production of DIMBOA at the highest concentrations of 200 plants 500 mL^−1^ (Supplementary Fig. 4). Similar effects of the root exudates and the presence of neighbors in a density-dependent manner suggest widespread occurrence of signaling components in the root exudates across different plant species.

**Fig. 4:**
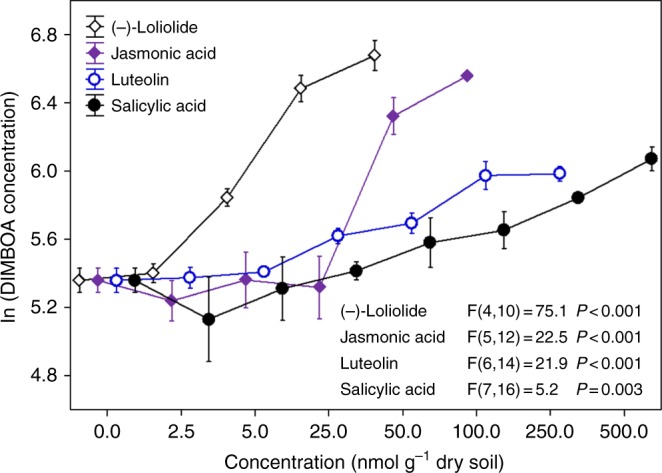
Induced effect of four potential signaling chemicals at diferent doses on the production of DIMBOA in wheat roots. The effect of concentration levels of the four potential signaling chemicals on the concentration of allelochemical DIMBOA was tested using linear model with chemical concentration as a fixed factor. Log_e_-transformed allelochemical DIMBOA were used as response variables. Values plotted are means (*n* = 3) ± standard errors (SE). The error bar denotes 1 SE

**Fig. 5:**
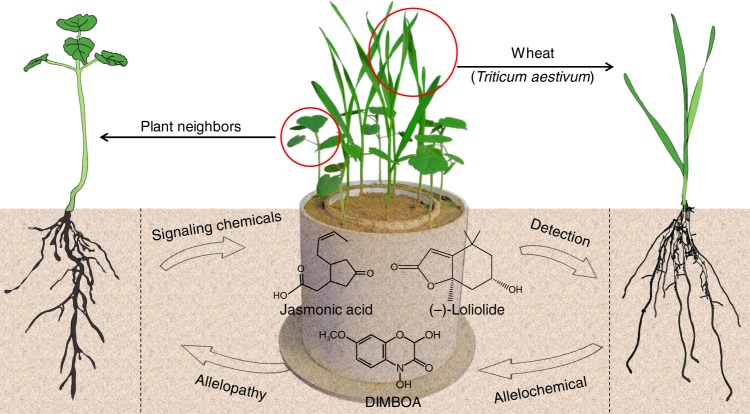
The belowground chemical interactions between wheat and plant neighbors. The root-secreted signaling chemicals (-)-loliolide and jasmonic acid from plant neighbors; The allelochemical DIMBOA response in wheat. Image and every element of this image were created by C.H.K., S.Z.Z., and Y.H.L

To identify which individual root exudate components could elicit DIMBOA production,a bioassay-guided fractionation approach was used (see Methods). Four potential chemical signals jasmonic acid (JA), salicylic acid (SA), (-)-loliolide and luteolin were identified (Supplementary Table 4). These presence of these potential signals was tested in all 100 heterospecifc neighbors. JA, SA and (-)-loliolide were found in wheat as well as all 100 heterospecifc neighbors, whereas luteolin was less common (Supplementary Table 5). These chemicals were also detected in the root exudates and rhizosphere soils of the subset of species tested (Supplementary Table 6). There was a significant positive correlation across species between neighbor-induced DIMBOA and their root-secreted (-)-loliolide concentrations (*r* = 0.76, *P* < 0.001), but such a significant correlation was not found for JA, SA or luteolin (Supplementary Fig. 5). Further, soil incubation showed that JA, SA, (-)-loliolide and luteolin all elicited the production of DIMBOA in a dose-dependent manner. Significant effects were observed for (-)-loliolide at a low concentration of 5 nmol g^−1^ dry soil and for JA at a medium concentration of 50 nmol g^−1^ dry soil. However, SA and luteolin required a higher concentration of over 100 nmol g^−1^ dry soil for elicitation (Fig. 4). In particular, the mixture of (-)-loliolide and JA at a low concentration greatly induced allelochemical response but the joint action was not observed in the mixture of (-)-loliolide and SA or luteolin (Supplementary Fig. 6). Furthermore, there were significant differences in mobility factor among the chemicals, with (-)-loliolide having the highest mobility in soil (Supplementary Fig. 7). Compared with SA and luteolin, (-)-loliolide and JA were more effective signaling chemicals to trigger the allelochemical response. Therefore, we suggest that wheat likely detects the presence of neighbors through root-secreted (-)-loliolide and JA in the soil, which can then initiate the release and production of DIMBOA, resulting in an induction of allelopathy (Fig. 5).

## Discussion

Plants can alter their growth and the production of secondary metabolites in response to the biotic and abiotic factors to which they are exposed in the environment^23,24^. Biochemical plasticity frequently occurs when one or more plant species occur together and interact. In particular, neighboring plants can profoundly affect plant biochemical composition through competition, allelopathy or both^28,35,36^. However, biochemical responses are strongly related to neighbor identity mediated through the presence of neighbor detection cues. Most studies have shown that neighbor identity influences plant biochemical responses in a species-specific fashion^14,25^. Consistent with our previous work^29,30^, we report here that allelopathic wheat produces DIMBOA in a density dependent response to neighboring plants and that root exudates are sufficient to induce this response. Allelopathic wheat appears to be able to detect the presence of plant neighbors when they occur early in development, and respond by increasing allelochemicals regardless of their neighbor identity. Wheat produces and releases benzoxazolinone allelochemicals, including DIMBOA and its glycosides, at early growth stages. Because of this, the hydrolysis and metabolic profiling of allelochemical DIMBOA from plants to soils is well-established^31–34^. In the current study, the increase in DIMBOA production was only two-fold or so. From a typical dose/response relationship, a two-fold increase in a toxin that is already present at a significant level may not be expected to have a substantial effect. However, a more dramatic effect might occur with joint action of other benzoxazolinone compounds of wheat.

Although the neighbor-induced allelochemical responses were density-dependent, these responses occurred primarily at intermediate neighbor densities. Plants growing with a high density of neighbors likely experienced an increase in competition-induced stress. Competition can alter plant allocation strategies, resulting in a shift from defense to growth^37,38^. Allelopathic plant species often increase allelochemical concentrations in response to low or intermediate levels of competition, but a decrease of allelochemicals at higher levels of competition where resource limitation may prevent such allocation^39^. Furthermore, the timing of plant exposure to competition may strongly mitigate allelochemical responsiveness. In the current study, induction of DIMBOA only occurred when wheat was exposed to competitors very early in development, with no response to later exposure. This suggests that there is a critical temporal window for detection of competition that also has large implications for species coexistence. In the crop-weed model system explored here, allelochemical induction early in development may aid the performance of plants exposed to competition. However, later germinating competitors will pose a much lower competitive risk because of their smaller size relative to the wheat, and therefore allocation to allelochemicals would be less beneficial to plant fitness. A similar strategy would be an advantageous response to competition in non-crop systems as well.

Although any stress caused by neighboring plants as a result of resource depletion or alteration of microbial communities might also induce an allelochemical response^40,41^, direct neighbor-induced allelochemical responses are critical to plant-plant interactions. Plant neighbor detection usually is mediated by direct contact or chemical release^4,11^. In belowground plant-plant signaling interactions, the detection of neighbors may occur through contact of root tips, soil microbes, especially common mycorrhizal networks^21,22^, or root-secreted signaling chemicals^14,42^. However, the mediators of belowground signaling interactions are still controversial due to inadequate methodology and our poor knowledge of the diversity of soil interactions. This study addressed the mechanism of belowground signaling through a series of experiments with and without root, mycorrhizal, and soil chemical segregation. This segregation-based approach clearly distinguished aboveground and belowground signaling interactions in generating allelochemical responses. Allelochemical production was induced by competitors with root segregation with both sizes of nylon mesh but not with complete segregation, where only aboveground signals would occur. Neighbor-induced allelochemical responses still occurred with the 0.45 μm mesh that would have segregated soil mycorrhizal hyphae but still allowed bacterial and chemical contact. Furthermore, allelochemical production was experimentally induced by the root exudates of neighboring plants, isolating the effects of chemicals from soil bacteria. These results indicatethat allelopathic wheat can detect neighboring plants purely through root-secreted signaling chemicals rather than via root contact, common mycorrhizal networks, or soil bacteria.

Plants may detect neighbors through plant volatiles as air-borne signals^10,11^, while root exudates drive belowground plant–plant signaling interactions^16,18^. Until recently, most studies on the signaling chemicals in plant–plant interactions have focused on jasmonic acid (JA) and salicylic acid (SA)^30,43–45^. JA and SA are two ubiquitous signaling chemicals which elicit the production of defensive plant metabolites against microbes, herbivores, or competitors^46,47^. However, information on other signaling chemicals involved in belowground plant–plant signaling interactions has remained scarce. Root-secreted strigolactones were described as signaling chemicals between parasitic plants and their hosts several decades ago^48^. Strigolactones also act as chemical signals for fungal symbionts and parasitic weeds in plant roots^49^. However, strigolactones do not appear to have a universal role in belowground plant-plant signaling interactions and there may be other, more specific, root-secreted signaling chemicals awaiting identification.

JA and SA may play some roles in belowground chemical communications. However, most studies have tested this with exogenous addition of JA and SA at arbitrary concentrations, rather than through quantification in the root exudates and rhizosphere soils^43,45^. A few studies have documented root-secreted JA and SA and their roles in rhizosphere signaling interactions^30,44^. In this study, besides the commonly studied JA and SA, (-)-loliolide and luteolin contributed to neighbor-induced allelochemical responses. In particular, (-)-loliolide and JA strongly induced allelochemical production at very low concentrations, whereas luteolin and SA required greater concentrations. Although these concentrations were greater when compared with the quantities of (-)-loliolide and JA as determined in the root exudates and rhizosphere soils from interacting weeds tested, we propose that such bioactive phytochemicals provided over a long time period at low concentrations might be able to induce substantial effects. Additionally, soil extractions would have diluted the chemical signals over large soil volumes while the concentrations of (-)-loliolide and JA detected would be locally much higher in intact soils. Furthermore, there was a joint action in the mixture of (-)-loliolide and JA at low concentrations. Thus, even if the actual concentration of (-)-loliolide and JA in soil was still substantially lower than the necessary concentration to elicit wheat allelochemical production, an effect would still be expected.

Similar to SA, phenolic luteolin is a signal in the resistance response of plant to microbes^50,51^. However, (-)-loliolide has never been reported in participating in any plant–plant or plant–microbe signaling interactions. More importantly, (-)-loliolide was detected in all of the 101 plant species tested while luteolin only occurred in 79 plant species. Actually, (-)-loliolide is the most ubiquitous lactone that occurs in many plant families and marine alga^52^. In the current study, (-)-loliolide was found in every monocot and dicot plant species tested and could be secreted from roots into rhizosphere soils. (-)-loliolide had a higher soil mobility and could easily be moved from the rhizosphere to bulk soil. Therefore, (-)-loliolide may be a ubiquitous, soil-borne signaling chemical that can trigger plant defensive responses in belowground plant–plant interactions.

Growth-inhibiting allelochemicals and signaling chemicals involved in plant–plant interactions profoundly affect the performance of plants, altering the consequences of intra-specific and inter-specific interactions in ecosystems^19,20,35^. It is now clear that plant-derived signaling chemicals contribute to plant neighbor detection and defensive responses. In general, chemicals produced by the competitor species that are xenobiotic to the focal plants might represent a better, species-specific signal, just like strigolactones as signaling chemicals of parasitic plants to the host^48,49^. However, (-)-loliolide and JA found in this study are ubiquitous signaling chemicals among plants, and are produced by the focal allelopathic wheat as well. In fact, signaling chemicals such as ethylene, JA, SA, and their formylates are ubiquitous plant–plant signals rather than xenobiotic chemicals that may indicate identity^10,11^. Plant neighbor detection and allelochemical response in generating coexistence heavily depended on neighbor density rather than neighbor identity, reminiscent of plant neighbor detection mediated in a dose-dependent fashion.

The (-)-loliolide or JA secreted from either conspecific or heterospecifc roots could elicit the production of allelochemicals and appears to be a signal for belowground competition, potentially common to all species. We speculate that variation in level of soil signaling chemicals produced across species has the potential to lead to variable responses in natural systems, though these chemical signals could still be general indicators of competition. We assume that there is an additional, species-specific signaling chemical that inhibits the induction of allelochemicals in conspecific interactions. This could be a much less common soil chemical, one more representative of the species identity and also much less likely to be detected by our methods which focused on chemicals that generated an allelochemical response. Fractionation-guided bioassays that focus on the inhibition of (-)-loliolide responses would be necessary to identify this soil signal.

The importance of belowground signaling interactions to plant neighbor detection and response strategies, as well as the mechanisms underlying such communications, has been a major focus of the science of plant interaction in recent years^14,16,42^. From a model system of allelopathic wheat and 100 interacting plant species, this study suggests that plant neighbor detection and allelochemical response mediated by root-secreted signaling chemicals are a general phenomenon. In particular, this study identified (-)-loliolide as a soil–borne chemical signal that could act to signify the presence of competitors and enhance allelochemical production.

In conclusion, the discovery of (-)-loliolide as a general soil-borne signaling chemical common to all the plants tested here, improves our understanding of plant neighbor detection and response strategies. Studies on the behavior of (-)-loliolide in the soil and its molecular biology, could lead to new insights into plant sensing and communication. Although a generic response to (-)-loliolide was detected here, there are likely to be additional signaling mechanisms that could allow species-specific responses. In addition, the ability of the signaling chemical to induce allelopathic responses in plants needs to be explored in other plant systems to test if they respond similarly to allelopathic wheat.

## Methods

### Plant materials and soils

Wheat produces and releases benzoxazinoids against various pests, most notably in affecting the growth of plant competitors. Among benzoxazinoids, 2,4-dihydroxy-7-methoxy-1,4-benzoxazin-3-one (DIMBOA) is a putative and dominant allelochemical^33,53^. Accordingly, a DIMBOA-rich winter wheat cultivar (Jing411) was used in this study and its seeds obtained from the Chinese wheat germplasm collection. Combining data from our previous study^29^ with new data, wheat plants were challenged with 100 plant species (Supplementary Table 2) which were selected on the basis of their occurrence and distribution in the local wheat industry or being ecologically relevant weeds and crops in agricultural ecosystems. Seeds were collected from local fields or obtained from germplasm collections in China. Soil for use in experiments was collected from the surface (0–10 cm) of a wheat field at the Shangzhuang Experimental Station of China Agricultural University (Beijing, China). Soil samples were air-dried, then sieved (2 mm mesh) to remove plant tissues. The soil is a Hapli-Udic Cambisol (FAO classification) with a pH of 6.52, an organic matter content of 1.65%, and a nutrient content of available N of 71.41 mg kg^−1^, available *P* of 70.12 mg kg^−1^, and available *K* of 94.51 mg kg^−1^.

### Wheat–neighbor interactions

Three experiments for wheat–neighbor interactions were carried out in plastic pots (11 cm diameter × 12 cm height) that contained a central cylinder (7.5 cm diameter, 12 cm height) where a barrier could be inserted (Supplementary Fig. 2). The experiments were conducted in a completely randomized design with three replicates for each treatment or control. Seeds of wheat and other plants were sterilized with 75% alcohol for 3 min, followed with 3% sodium hypochlorite for 12 min. Sterilized seeds were rinsed with distilled water and then transferred to Petri dishes (9 cm diameter) with moistened filter paper and were pre-germinated at 28 °C in the dark.

The first experiment investigated the allelochemical response of wheat to eight commonly interacting weeds (*E. indica, D. sanguinalis, A. theophrasti*, *B. frondosa, L. perenne, A. fatua, A. japonicus*, and *A. tauschii*) at different densities. Pre-germinated seeds of wheat and neighbor pairs were each sown into the plastic pot containing 800 g of soil with no barrier. Five wheat seeds were spaced uniformly in the central cylinder of each pot, while neighbor seeds were sown surrounding the wheat. Proportions of wheat to eight common neighbor species ranged from 5:1 to 5:10 per pot (wheat:neighbor), while trials between wheat and the remaining 92 species (Supplementary Table 2) were conducted at 5:8 and/or 5:5 mixture proportions. The plant:competitor ratios were found to generate sufficient responses (Fig. 1). Pots with wheat monocultures in the same planting patterns and mixture proportions served as the controls for all analyses.

A second experiment was run to determine the influence of time of association on allelochemical response of wheat to eight commonly interacting weeds at the 5:5 mixture. A series of experimental pots containing 800 g of soil with no barriers and were planted with five wheat seeds sown into the central cylinder and five neighbor seeds sown in the surrounding area. Plantings occurred simultaneously, neighbors planted after the wheat emerged, or wheat planted after the neighbors had emerged. Again, pots with wheat monocultures for each group served as the controls.

The third experiment evaluated allelochemical response of wheat to eight commonly interacting weeds with varying levels of belowground segregation (Supplementary Fig. 2). A series of pots containing 800 g of soil had central cylinders that were: open (full contact), covered with 30 μm mesh (prevented penetration of roots but allowed chemical and microbial interactions), covered with 0.45 μm mesh (prevented penetration of both roots and common mycorrhizal hyphae but allowed chemical and bacterial interactions) or covered with plastic film (complete separation). As previously described, wheat and neighbor pairs at 5:8 and/or 5:5 proportions were sown simultaneously in the pots with or without belowground segregation. Monocultures of wheat-wheat (5:5 or 5: 8) in a pot for each group or treatments served as the controls.

All pots from the experiments described above were placed in a greenhouse with 20–30 °C night and daytime temperatures and 65–90% relative humidity, watered daily and their positions randomized once a week. Any emerging plants, other than wheat or sown neighbor species were hand removed as soon as they were detected. Seedlings were harvested after 4 weeks and separated carefully into wheat and neighbor plants. In addition, rhizosphere soils from the third experiment were collected by shaking soil off from roots from treatments separated by nylon mesh according to the procedure of Guo et al.^54^. Plant and soil samples were taken for the quantification of allelochemical DIMBOA and signaling chemicals as described in the quantitative analysis section below.

### Potential signaling components from root exudates

To obtain substantial amounts of potential signaling components, a larger scale collection of the root exudates was carried out with a modified continuous root exudate-trapping system^55^ in a greenhouse (Supplementary Fig. 8). Three-thousand seedlings of each of eight commonly interacting weeds at the 3-leaf or 5-leaf stage were transplanted into culture containers (2 m × 1 m × 0.3 m) with 1/2 Hoagland’s solution. Chemical trapping was done within a column (5 cm × 50 cm) with 1000 g Amberlite XAD-4 resin (Sigma-Aldrich Co., St. Louis, MO, USA) packed into it. This was connected to the reservoir with a pump and the culture solution was circulated for 2 h though it once a day. After 10 days, the column was detached. The resin was continuously washed with distilled water for 24 h to purge inorganic ions and carbohydrates. The resin was then eluted with methanol. After filtration, the exudates were evaporated to dryness in vacuo by using a rotary evaporator at ambient temperature.

The dried root exudates (100 g) were successively partitioned three times with petroleum ether (PE), followed by methylene chloride (CH_2_Cl_2_). The CH_2_Cl_2_ extract was concentrated, and subjected to silica gel column chromatography (5 cm × 80 cm) by eluting stepwise with a series of mixtures of PE, CH_2_Cl_2_ and MeOH (10:0:0, 8:2:0, 6:4:0, 4:6:0, 2:8:0, 0:10:0, 0:9:1, 0:8:2, 0:7:3, 0:6:4, 0:5:5, and 0:0:10, v/v/v). The fractions were screened using a bioassay-driven fractionation approach^56^ as described in the soil incubation section below, resulting in four fractions that induced the production of allelochemical DIMBOA in wheat. The first fraction was further purified by silica gel column chromatography (2.5 cm × 40 cm) with a mixture of CH_2_Cl_2_ and MeOH (5:5, v/v), and yielded luteolin. The second fraction was further purified by Sephadex LH-20 (20–150 μm, 1 cm × 25 cm) with MeOH, resulting in liquid jasmonic acid (JA). The third fraction was further purified by ODS (YMC 120 A 50 μm, 1 cm × 25 cm) with H_2_O containing increasing amounts of MeOH to obtain a green solid that was recrystallized with CH_2_Cl_2_ to yield (-)-loliolide. The fourth fraction was further purified by silica gel column chromatography (2.5 cm × 40 cm) with a mixture of CH_2_Cl_2_ and MeOH (3:7, v/v), resulting a crude solid that was recrystallized with *n*-hexane-MeOH (4:6; v/v) mixture and gave salicylic acid (SA). All four chemicals, JA, SA, (-)-loliolide and luteolin, were identified by spectroscopic analysis (Supplementary Table 4) and chromatographic co-elution with authentic standards (Sigma-Aldrich Co., St. Louis, MO, USA).

### Soil incubation

Induction activities of root exudates and their signaling components on the production of DIMBOA were verified using a pot-culture study. The root exudates of wheat and eight commonly interacting weeds were collected in a hydroponic experiment. Fifty, one or two hundred seedlings at the 3-leaf stage for each species were respectively inserted into holes in a Styrofoam float and transplanted into a hydroponic container with 1/2 Hoagland’s solution (500 ml). The container was placed in a sterile environmental chamber at 28 ± 1 °C with a 12 h photoperiod. The hydroponic solution in the container was kept at a constant level by adding distilled water daily. After 7 days the hydroponic solution was filtered with sterile filter papers, and the filtrate was collected to yield the root exudates.

Ten wheat seeds were sown into a series of 5 cm × 5 cm plastic pots with 100 g of soil. At the 2-leaf stage these were thinned to fivee plants per pot. The root exudates described above, one of the fractions or mixtures and potential signaling components (i.e., jasmonic acid, salicylic acid, (-)-loliolide or luteolin) that were isolated and identified as describe in the section above, were each added to the treated pots at different concentrations. Control pots received distilled water only. All treatments and controls were replicated three times and were placed in an environmental chamber with a temperature of 25 °C and 65–90% relative humidity. The pots were randomly sampled at 6 h after soil-incubation. This sampling time was based on a significant increase in wheat DIMBOA observed in an incubation experiment involving the addition of weed root exudates^29^. Wheat seedlings were collected for the quantification of DIMBOA as described in the quantitative analysis section below.

### Soil thin layer chromatography (TLC)

Soil TLC was performed by a combination of two methods^57,58^. Soils as described above were ground and sieved to 125 μm. The soil was suspended in a dioxane/water (1:1, v/v) solvent to make a slurry which was then spread as a 0.7 mm thick layer on a 10 cm × 20 cm glass plate. The plates were air-dried at 20–25 °C and stored in a desiccating chamber until used for chromatographic tests. Four potential signaling chemicals, JA, SA, (-)-loliolide or luteolin were each sampled with a microsyringe at 2.5 cm from the bottom edge of the plates. Distilled water in sampling served as the control. After the spots had been deposited, the plates were allowed to develop in a closed glass chamber using distilled water as solvent. A sheet of filter paper dipping into the developing water fed water continuously to the substrate at the base of the plate, thus leading to a relatively uniform flow. During development with water, the whole device was held in a horizontal position. Water migration occurred at a distance 17.5 cm from the baseline. The migration lasted between 1 h and 5 h depending on the samples. The plates were dried at 20–25 °C, and the soil layer of drying the developed TLC plates with three replicates for each chemical was cut into segments of 1.5 cm each. To avoid microbial degradation and transformation, chemical residue in each segment was quantified immediately by ultra performance liquid chromatography (UPLC) described below. Mobility factor (Rf) value of each chemical was calculated according to the formulae *R*f = *∑R*_i_ × *M*_i_ /*R*_w_ × *∑M*_i_, where, *R*_w_ was remove distance of water from start point, i was number of segments, R_i_ was distance of segment *i* from start point, *M*_i_ was chemical content in segment *i*^59^.

### Quantitative analysis of allelochemical and signaling chemicals

Quantification of the allelochemical DIMBOA was performed by liquid extraction/solid-phase extraction, followed by high-performance liquid chromatography (HPLC). The wheat tissues (roots or shoots) were freeze-dried and ground with liquid nitrogen. Then 500 mg of the resulting powder was homogenized with 50 ml of 25% aqueous MeOH and extracted by ultrasonic oscillator for 30 min at a temperature of 25 °C. The extract was filtered, and the filtrate was evaporated to dryness individually under nitrogen gas. Dry residues were dissolved in 2.5 ml of 0.05% acetic acid (HOAC) in a MeOH-H_2_O mixture (60:40 v/v) and then loaded onto reverse-phase C_18_ Sep-Pak cartridges (Waters Co., Milford, MA, USA) equilibrated with water, which eluted with acidified MeOH (1% HOAC) and then MeOH. The MeOH fraction was concentrated with nitrogen gas to a final volume of 100 μl. The concentrated samples were subsequently subjected to an HPLC-1260 instrument (Agilent, Palo Alto, CA, USA) equipped with a C_18_ reverse-phase column (Hypersil 4.6 mm × 150 mm, 5 μm) and a diode array UV detector at 280 nm. Elution was performed with a mixture of 0.5% acetic acid and MeOH (70:30, v/v) at a constant flow rate of 1.0 ml min^−1^ at 40 °C. The peak of DIMBOA was identified by its retention time (9.8 min) and coelution with an authentic standard (Regenstauf, Germany). DIMBOA was quantified by regression analysis of the peak areas against standard concentrations (limit of quantification, 10 μg g^−1^; recovery rates at concentrations of 200–1500 μg g^−1^, 66.5–82.4%).

Four potential signaling chemicals JA, SA, (-)-loliolide and luteolin were quantified by ultra-performance liquid chromatography coupled with tandem mass spectrometry (UPLC-MS/MS). The plant tissues, root exudates or rhizosphere soils were each freeze-dried and ground with liquid nitrogen. An amount of 250 mg of the resulting powder was extracted with 10 ml of MeCN (acetonitrile)-H_2_O-HOAC mixture (90:9:1, v/v/v), vortexed for 5 min at 25 °C and then NaCl was added and immediately vortexed vigorously for 1 min. After the solution was centrifuged at 2800×*g* for 10 min, the supernatant was filtered with a 0.22 μm nylon syringe filter (Sterlitech, Kent, WA, USA). Analyses of four chemicals were carried out on a triple–quadrupole mass spectrometer (TQD, Waters Co., Milford, MA, USA) equipped with an electrospray ionization (ESI) source operating in positive mode for (-)-loliolide and luteolin and negative mode for JA and SA. Instrument control and data acquisition were performed using MassLynx software (version 4.1). Chromatographic separation was performed using an Acquity UPLC-BEH C_18_ column (50 mm × 2.1 mm, 1.7 μm) at 40 °C. The injection volume was 5 μl. The elution gradient was carried out with a binary solvent system consisting of 0.2% HOAC in H_2_O (solvent A) and MeCN (solvent B) at a constant flow rate of 0.3 ml min^−1^. Simultaneous separations were completed using a gradient elution of 0.0 min/90% A, 2 min/10% A, 3.0 min/10% A, 4 min/90% A, and 5.0 min/90% A. Separation and stabilization were achieved in 5.0 min. The typical conditions were capillary voltage, 3.0 kV; source temperature, 120 °C; and desolvation temperature, 350 °C. The cone and desolvation gas were set at flow rates of 50 and 600 l h^−1^, respectively. Multi-reaction monitoring (MRM) mode was operated for each chemical. All parameters for the MRM transitions, cone voltage and collision energy were optimized to obtain the highest sensitivity and resolution. For JA: retention time, 1.60 min; parent ion, *m*/*z* 209; product ion, *m*/*z* 58.9; cone voltage, 35 V. For SA: retention time, 1.40 min; parent ion, *m*/*z* 136.8; product ions, m/z 92.9; cone voltage, 26 V. For (-)-loliolide: retention time, 1.25 min; parent ion, *m*/*z* 197; product ion, *m*/*z* 178.7; cone voltage, 23 V. For luteolin: retention time, 1.35 min; parent ion, *m*/*z* 287; product ion, *m*/*z* 153; cone voltage, 20 V. Quantification of four chemicals was each conducted by the addition method with authentic standards. Limit of detection (LOD) for the chemicals is the concentration that produces *S*/*N* (signal-to-noise ratio) = 3, estimated from the chromatogram corresponding to the lowest concentration used in the calibration, whereas limit of quantification (LOQ) is defined based on S/N = 10. The LOD were estimated at 0.006 nmol g^−1^ for loliolide, 0.004 nmol g^−1^ for luteolin, 0.004 nmol g^−1^ for JA and 0.006 nmol g^−1^ for SA. The LOQ were estimated at 0.02 nmol g^−1^ for loliolide, 0.01 nmol g^−1^ for luteolin, 0.01 nmol g^−1^ for JA and 0.02 nmol g^−1^ for SA. The recoveries of the four chemicals ranged in 86.2–102.6% (RSD, 4.56–10.88) at the spike level of 0.01–150 nmol g^−1^.

### Data analysis

For the survey of 100 species, data were presented as means ± standard error (SE) from each of independent experiments with three replicates. The data were analyzed using Student’s *t*-test or analysis of variance (ANOVA). Tukey post-hoc tests were used for multiple comparisons when ANOVA terms were significant using SPSS 16.0 for Windows (SPSS Inc. Chicago, Illinois, USA). Regression lines were fitted as linear or quadratic model results using Sigmaplot 10.0 (Systat Software Inc. San Jose, California, USA). A simple Pearson’s correlation was used to test whether the allelochemical DIMBOA induction across samples correlated with signaling components of the root exudates also using SPSS. While we did not control for multiple comparisons in the response survey of 100 species, we calculated binomial probabilities for obtaining the number of significant individual tests. To provide a single overview of this survey, we calculated Ln-transformed response ratios relative to the wheat:wheat treatment in each species for both the DIMBOA concentration and biomass of roots and shoots. From these 100 ratios, we present means ±95% confidence intervals as a measure of effect size.

## Electronic supplementary material

Supplementary Information

## Data Availability

All relevant data supporting the findings of this paper are available from the corresponding author on request.
