# Supplementary material for: Plant neighbor detection and allelochemical response are driven by root-secreted signaling chemicals
Source: Nat Commun. 2018 Sep 24;9:3867. doi: 10.1038/s41467-018-06429-1 (PMC6155373; doi:10.1038/s41467-018-06429-1)
Supplement: Supplementary file 1 — Supplementary Information [file 41467_2018_6429_MOESM1_ESM.pdf]

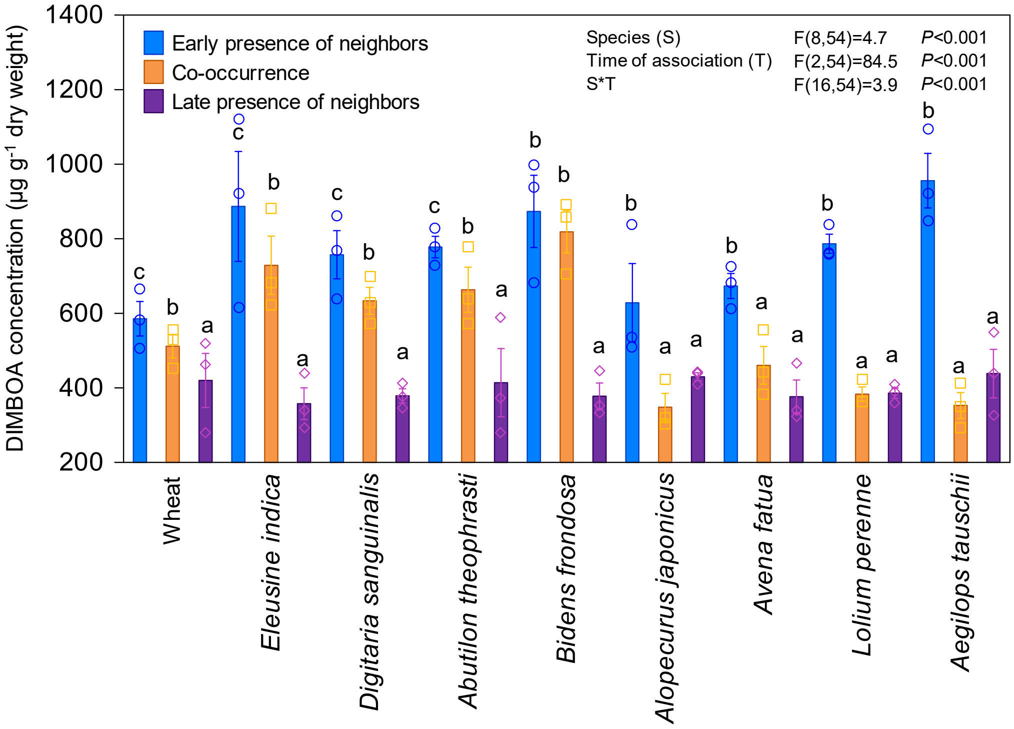

Supplementary Fig. 1 Variation in the concentration of allelochemical DIMBOA of wheat roots in response to neighbors exposed at different sequences. The effect of the time of association and species identity on the concentration of allelochemical DIMBOA was tested using linear model with time of association and species as fixed factors. Tukey post hoc tests were calculated between treatments of time of association. Values plotted are means (n=3) ± standard errors (SE). Columns with different letters indicate significant difference among time of association within each species at $P<0.05$. The error bar denotes 1 SE.

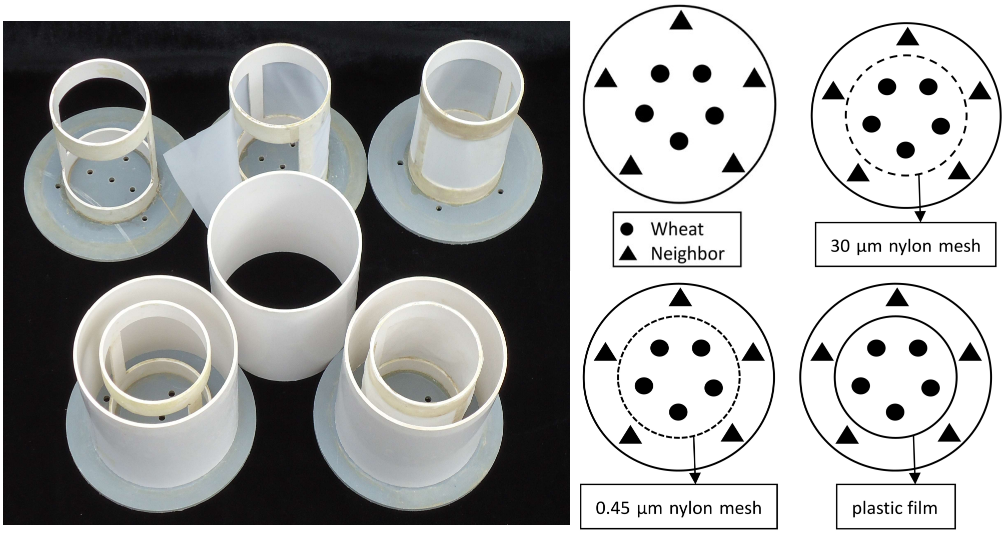

Supplementary Fig. 2 Schematic diagram of experimental design with belowground segregation in wheat-neighbor interactions. Photographic image was created by Y.H.L.

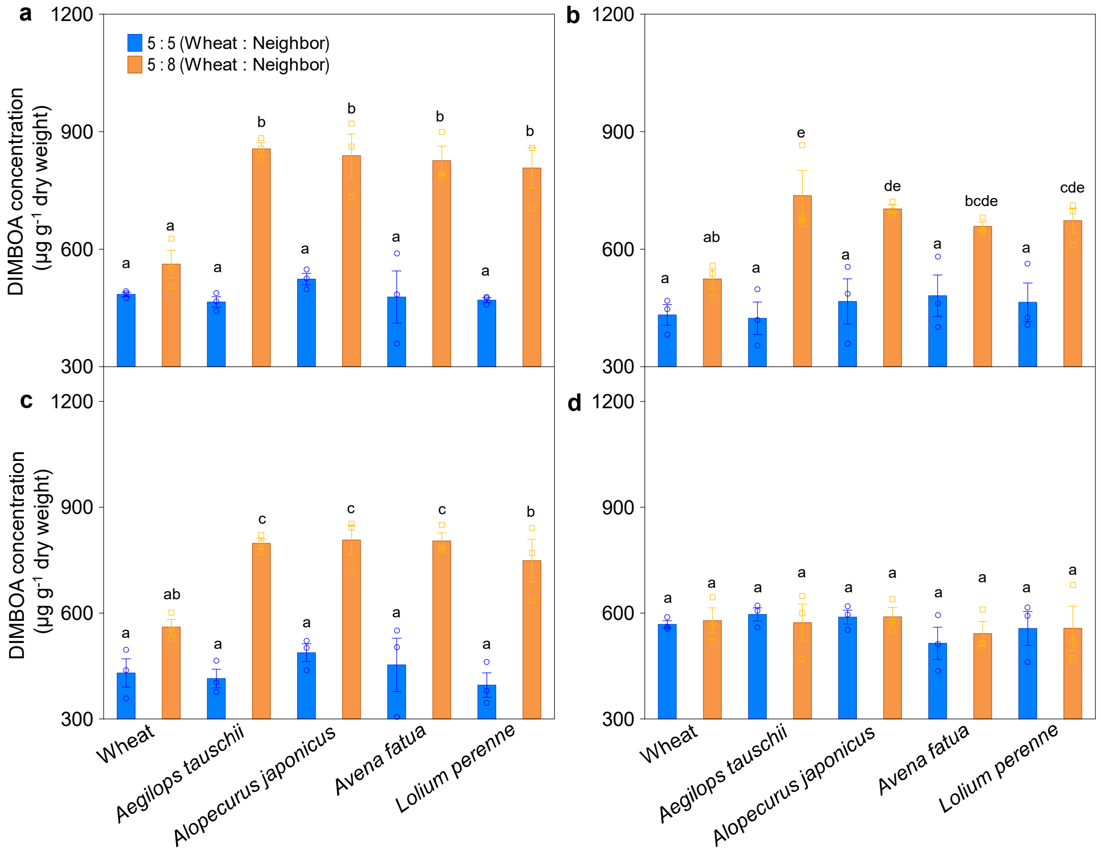

Supplementary Fig. 3 A comparison of the DIMBOA concentration in roots of wheat grown in the presence of neighbors at 5:5 and 5:8 proportions with various levels of belowground segregation. **a** without segregation; **b** segregation with 30 μm nylon mesh; **c** segregation with 0.45 μm nylon mesh; **d** segregation with plastic film. The effect of segregation type and mixture proportion and species identity on the concentration of allelochemical DIMBOA in roots was tested using linear model with segregation type, mixture proportion and species as fixed factors. Tukey post hoc tests were used to analyze the difference between 5:5 and 5:8 mixture proportions among the four levels of belowground segregation. F-values, *P*-values and residual degrees of freedom are shown for ANOVAs in Supplementary Table 3. Values plotted are means (n=3) ± standard errors (SE). Columns with different letters indicate significant difference among mixture proportion and species within each belowground treatment at *P*<0.05. The error bar denotes 1 SE.

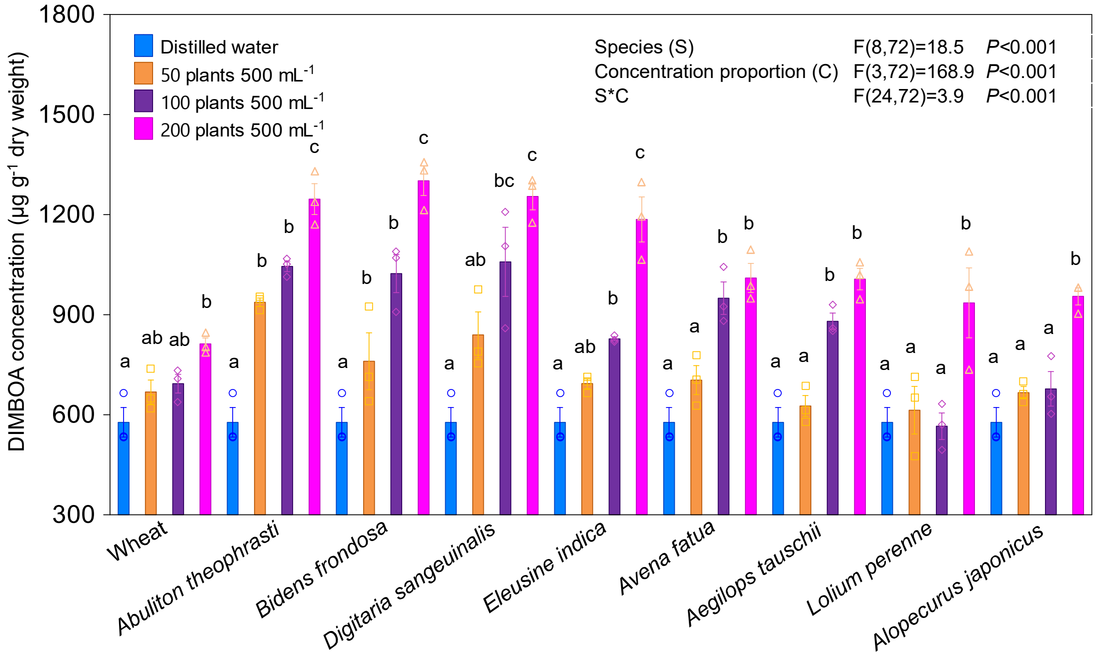

Supplementary Fig. 4 The DIMBOA production of wheat roots exposure to the root exudates from neighbors at varying concentrations. The effect of concentration proportion and species identity on the concentration of allelochemical DIMBOA was tested using linear model with concentration proportion and species as fixed factors. Tukey post hoc tests were calculated between treatments of concentration proportion. Values plotted are means (n=3) ± standard errors (SE). Columns with different letters indicate significant difference among different treatments within each species at $P < 0.05$. The error bar denotes 1 SE.

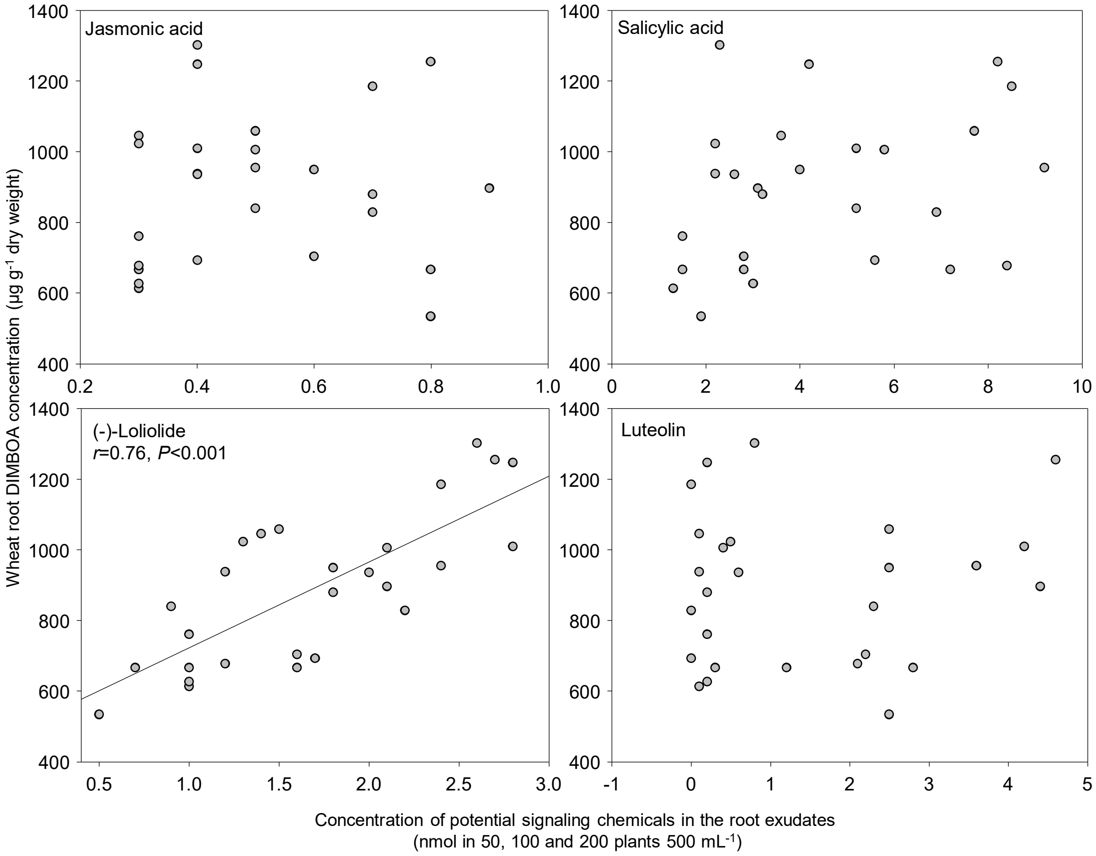

Supplementary Fig. 5 The correlation across wheat and eight commonly interacting species between neighbor-induced DIMBOA and their root-secreted four potential signaling chemicals concentrations.

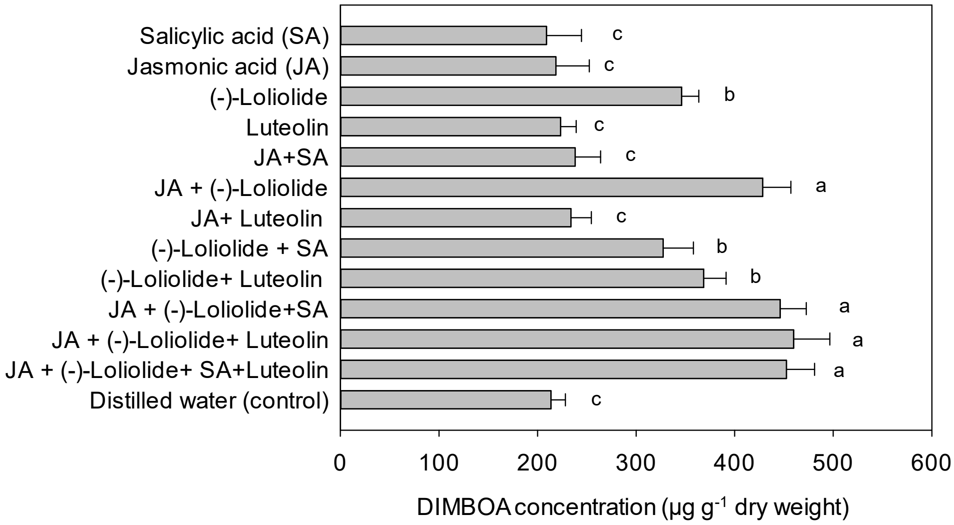

Supplementary Fig. 6 Joint action of potential signaling chemicals (5 nmol g^{-1} dry soil) at 1:1 mixture proportion on DIMBOA production in wheat roots. Values plotted are means (n=3) ± standard errors (SE). Columns with different letters indicate significant difference among chemicals and their mixtures at $P<0.05$, one-way ANOVA, followed by Tukey's honestly significant difference tests. The error bar denotes 1 SE.

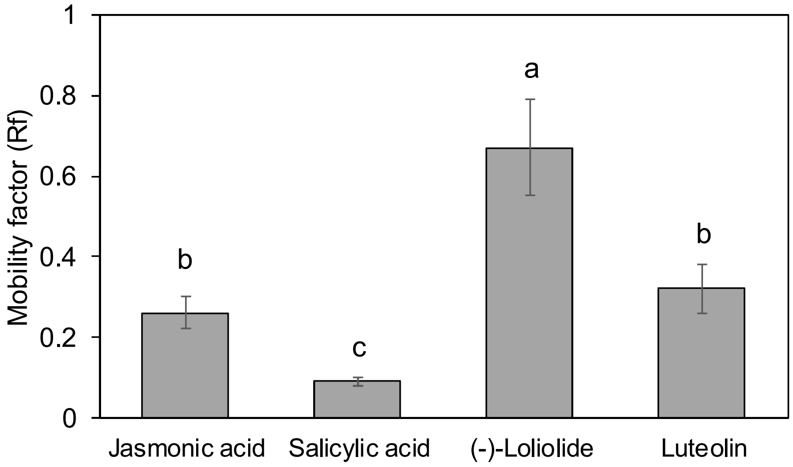

Supplementary Fig. 7 The mobility of potential signaling chemicals in soil. Values plotted are means (n=3) ± standard errors (SE). Columns with different letters indicate significant difference among chemicals at P<0.05, one-way ANOVA, followed by Tukey's honestly significant difference tests. The error bar denotes 1 SE.

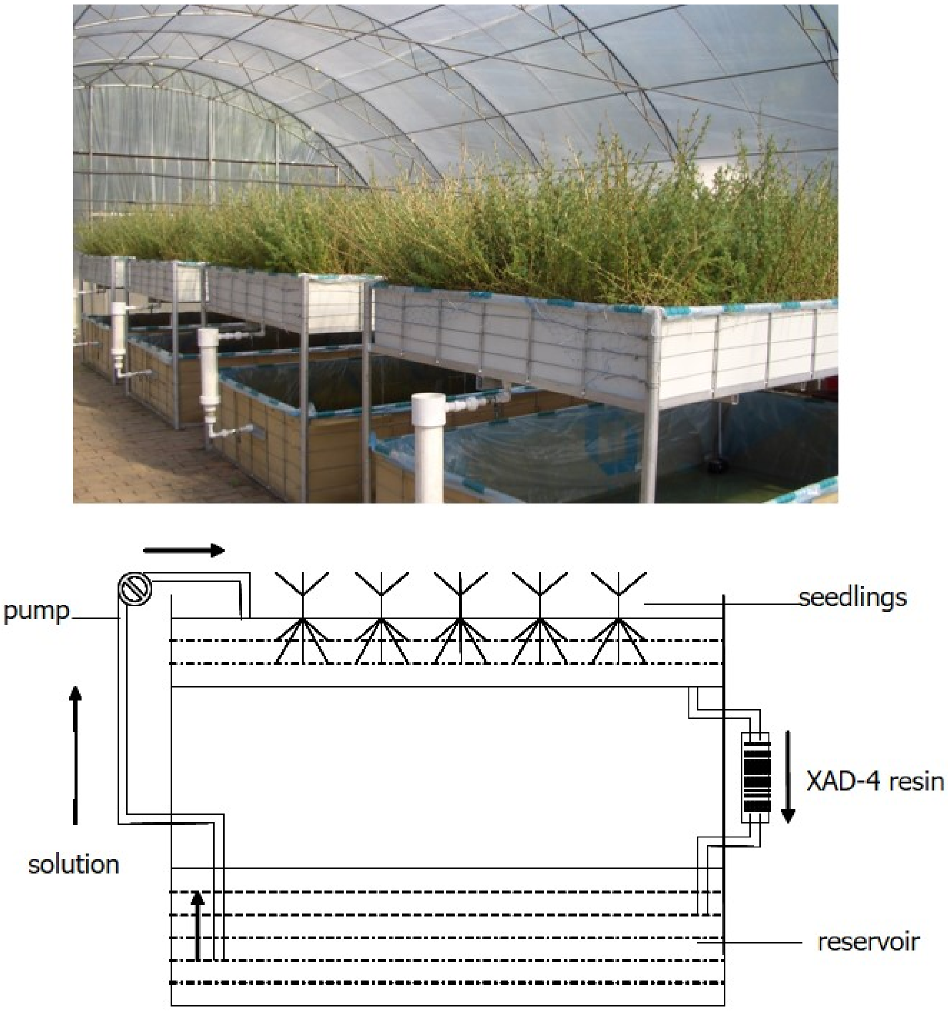

Supplementary Fig. 8 An automated continuous trapping system for a large scale collection of the root exudates. Photographic image was created by C.H.K.

Supplementary Table 1 Significance of polynomial terms for Fig. 1

**Plant species**	**DIMBOA concentration**			
	**Effect**	***df***	**F**	***P***
Eleusine indica	Neighbor density (D)	1, 54	4.7	0.035
	D^2	1, 54	19.5	<0.001
	Species (S)	1, 54	3.6	0.065
	D×S	1, 54	2.6	0.113
	D^2×S	1, 54	16.8	<0.001
Lolium perenne	Neighbor density (D)	1, 54	179.0	<0.001
	D^2	1, 54	2.5	0.12
	Species (S)	1, 54	10.5	0.002
	D×S	1, 54	0.0	0.936
	D^2×S	1, 54	3.9	0.054
Digitaria sanguinalis	Neighbor density (D)	1, 54	7.3	0.01
	D^2	1, 54	18.3	<0.001
	Species (S)	1, 54	5.7	0.021
	D×S	1, 54	6.7	0.012
	D^2×S	1, 54	8.7	0.005
Avena fatua	Neighbor density (D)	1, 54	17.0	<0.001
	D^2	1, 54	6.5	0.013
	Species (S)	1, 54	10.1	0.002
	D×S	1, 54	1.2	0.282
	D^2×S	1, 54	7.5	0.008
Abutilon theophrasti	Neighbor density (D)	1, 54	14.9	<0.001
	D^2	1, 54	3.4	0.07
	Species (S)	1, 54	5.0	0.03
	D×S	1, 54	1.5	0.223
	D^2×S	1, 54	6.2	0.015
Alopecurus japonicus	Neighbor density (D)	1, 54	97.8	<0.001
	D^2	1, 54	1.7	0.195
	Species (S)	1, 54	5.6	0.022
	D×S	1, 54	0.6	0.441
	D^2×S	1, 54	2.5	0.122
Bidens frondosa	Neighbor density (D)	1, 54	45.5	<0.001
	D^2	1, 54	15.5	<0.001
	Species (S)	1, 54	4.4	0.041
	D×S	1, 54	0.2	0.64
	D^2×S	1, 54	11.3	0.001
Aegilops tauschii	Neighbor density (D)	1, 54	70.7	<0.001
	D^2	1, 54	3.1	0.081
	Species (S)	1, 54	7.7	0.007
	D×S	1, 54	3.5	0.067
	D^2×S	1, 54	0.4	0.512

Supplementary Table 2 Allelochemical DIMBOA concentration and biomass of wheat in the presence of 100 plant neighbors at 5:8 and/or 5:5 mixture proportions (Neighbor species arranged in decreasing order of DIMBOA concentration in roots at the 5:5 wheat: neighbor ratio)

Plant species	Mixture proportion	DIMBOA concentration （μg g^{-1} dry weight）		Biomass （mg dry weight $plant^{-1}$）	
	Wheat : neighbor	Root	Shoot	Root	Shoot
Triticum aestivum (Control)	5:5	769.6±41.3	293.1±12.5	17.0±0.3	45.4±2.3
	5:8	740.5±26.3	225.1±17.8	17.8±0.3	44.9±1.4
Raphanus sativus	5:5	1751.4±193.3*	333.17±22.6	16.7±1.3	40.9±0.5
Glycine max	5:5	1559.1±200.6*	383.2±44.6*	16.6±2.4	42.4±2.6
Kummerowia striata	5:5	**1506.1±47.0***	481.5±27.8*	17.6±1.8	44.0±3.1
Abutilon theophrasti	5:5	**1418.7±254.3***	417.5±24.9*	17.3±0.9	44.4±1.9
Plantago asiatica	5:5	**1396.8±44.9***	437.3±34.7*	16.8±0.4	47.0±1.9
Medicago sativa	5:5	1298.4±96.1*	389.3±59.9	18.6±0.7	46.6±2.4
Vigna radiata	5:5	1287.3±134.9*	455.4±74.7*	17.2±1.6	42.9±0.8
Kochia scoparia	5:5	**1277.1±95.5***	301.6±11.6	18.8±0.7	46.5±3.4
Daucus carota	5:5	1255.9±56.8*	323.3±0.3	17. 5±1.0	46.3±2.6
Orychophragmus violaceus	5:5	1255.9±101.9*	314.5±4.4	17.2±2.3	45.4±2.6
Polypogon fugax	5:5	**1217.4±43.9***	317.6±25.3	17.2±1.0	46.8±3.0
Digitaria sanguinalis	5:5	**1202.4±131.1***	418.6±34.2*	18.1±1.6	47.6±3.8
Cucumis sativus	5:5	1180.4±73.9*	279.9±32.7	18.7±0. 8	47.1±1.8
Eleusine indica	5:5	**1178.4±76.4***	279.5±7.9	15.3±0.6	48.1±2.8
Fagopyrum tataricum	5:5	**1169.9±54.8***	289.6±13.5	16.7±0.8	48.3±2.5
Lepidium apetalum	5:5	**1160.3±133.2***	297.9±41.4	15.6±0.5	44.3±0.7
Bidens frondosa	5:5	**1142.7±95.3***	407.9±45.1*	17.9±0.6	45. 9±1.3
Phytolacca acinosa	5:5	1130.4±88.6*	295.7±18.2	17.3±1.0	47.6±1.2
Vicia sativa	5:5	**1115.8±123.5***	289.3±25.8	15.8±2.6	45.4±2.5
Spinacia oleracea	5:5	1108.2±75.0*	281.3±26.1	17.4±0.9	47.6±2.9
Phragmites australis	5:5	1104.9±79.5*	315.3±3.9	18.0±1.0	49.0±0.7
Chrysanthemum coronarium	5:5	1099.9±88.5*	313.2±26.5	17.4±0.7	49.5±1.4
Hibiscus trionum	5:5	**1083. 9±90.8***	343.4±20.2	17.6±0.3	44.9±0.9
Cucurbita moschata	5:5	1081.1±47.1*	301.1±9.5	17.5±0.9	43. 6±2.2
Descurainia sophia	5:5	1080.9±81.74*	366.6±12. 7*	16.8±0.1	44.8±0.5
Vigna unguiculata	5:5	1076.8±10.4*	307.3±15.3	15.1±0.9	39.2±2.2*
Stipa krylovii	5:5	1071.3±71.0*	302.6±4.4	17.7±0.7	48.7±0.8
Aeschynomene indica	5:5	1063.7±72.3*	377.1±6.6*	16.2±0.8	43.9±0.6
Rumex patientia	5:5	1052.9±81.1*	316.9±16.7	17.2±0.1	44.8±1.2
Chenopodium serotinum	5:5	**1045.3±50.0***	323.1±21.1	14.8±0.3*	47.9±1.4
Cirsium setosum	5:5	1038.1±57.6*	294.0±28.3	18.0±0.3	46.8±0.6
Ageratum conyzides	5:5	1036.5±77.4*	341.1±32.6	16.5±1.2	44.6±1.5
Coriandrum sativum	5:5	1034.1±66.0*	301.0±5.6	16.8±0.7	45.8±1.9
Chenopodium album	5:5	**1021.1±92.1***	326.7±29. 6	16.3±2.8	42.5±1.8

Chloris virgata	5:5	**987.1±63.7***	321.8±15.1	17.7±0.9	51.2±1.8
Arctium lappa	5:5	962.7±54.1*	281.9±6.6	16.7±1.2	45.0±2.5
Cynodon dactylon	5:5	952.8±29.2*	279.5±29.9	17.1±0.6	44.4±2.5
Lactuca sativa var. ramose	5:5	887.1±33.0*	277.5±11.1	17.1±0.5	48.1±1.9
Dactyloctenium aegyptium	5:5	1040.8±126.3	316.3±18.3	17.8±1.3	44.1±1.3
	5:8	1033.8±36.9*	311.5±22.2	16.1±0.4	46.6±0.9
Echinochloa crusgalli	5:5	**1035.4±122.2**	292.4±39.4	16.4±2.3	49.1±2.3
	5:8	1017.8±47.4*	314.2±18.3	17.9±0.8	45. 8±1.6
Avena fatua	5:5	**985.1±81.1**	344.0±6.1	16.8±1.3	48.5±3.4
	5:8	1025.2±73.6*	355.1±58.9	18.7±0. 6	51.9±0.8*
Senna tora	5:5	968.7±118.2	287.7±9.4	17.5±1.3	46.5±2.6
	5:8	995.8±63.4*	271.8±11.3	17.9±0.4	46.9±0.9
Alopecurus aequalis	5:5	**964.4±120.2**	323.4±58.8	16.9±1.2	44.7±2.4
	5:8	2184.2±107.2*	345.1±14.0	18.8±1.1	50.3±1.8
Silene conoidea	5:5	**962.9±117.2**	315.8±17.6	16.4±0.5	45.0±0.6
	5:8	938.7±43.6*	328.2±1.7	18.8±0.7	50.2±1. 6
Phaseolus vulgaris	5:5	957.1±21.7	280. 5±49.2	16.2±0.3	43.3±0.4
	5:8	1035.8±109.6*	421.1±7.3*	12.5±0.9*	39.2±0.4*
Lactuca sativa	5:5	940.1±193.1	314.2±74.9	18.3±0.7	48.4±2.4
	5:8	1098.4±93.3*	333.5±25.7	15.3±0.3	44.89±1.5
Artemisia integrifolia	5:5	919.7±99.6	326.4±12.6	17.5±0.3	50.3±1.1
	5:8	1010.8±66.4*	278.5±14.5	17.8±0.1	46.2±1.0
Senna occidentalis	5:5	910.0±81.3	298.4±14.3	18.5±0.3	50.7±1.5
	5:8	1003.0±34.7*	316.3±11.9	19.3±1.1	43.9±2.0
Alopecurus japonicus	5:5	**897.6±154.9**	409.8±46.1*	14.9±0.7*	43.1±2.2
	5:8	996.7±96.9*	305.5±4.1	21.5±0.4*	53.6±0.7*
Helianthus annuus	5:5	877.1±115.5	299.6±13.1	16.7±0.5	43.6±1.8
	5:8	1560.5±123.1*	274.4±12.7	16.7±0.2	44.6±1.6
Lagopsis supina	5:5	**859.2±36.2**	344.1±17.0	18.5±1.7	47.9±1.8
	5:8	967.9±33.5*	280.8±3.7	21.3±0.5*	51.1±1.9
Gossypium hirsutum	5:5	838.3±99.2	301.9±23.7	18.0±1.0	42.9±0.5
	5:8	1029.1±61.2*	289. 8±16.6	18.2±0.4	48.8±2.3
Calystegia hederacea	5:5	**830.1±165.7**	280.2±33.8	16.1±2.5	44.1±2.1
	5:8	1335.4±111.2*	298.1±15.9	19.3±1.4	45.1±1.4
Fagopyrum esculentum	5:5	825.9±25.3	283.9±16.1	15.9±0.8	44.9±1.9
	5:8	1167.6±52.7*	265.7±17.2	16.9±0.4	46.3±0.7
Veronica didyma	5:5	**804.4±39.9**	327.4±10.4	16.7±0.8	44.9±1.9
	5:8	1134.1±62.6*	283.68±2.6	19.9±0.6*	52.7±3.3*
Vaccaria segetalis	5:5	**794.6±51.3**	299.3±9.9	17.2±0.4	49.6±2.5
	5:8	1156.3±90.9*	294.5±17.0	19.5±0.6*	52.2±2.5*
Eclipta prostrata	5:5	794.1±186.5	335.8±13.2	16.1±0.8	39.3±2.6
	5:8	1139.7±79.9*	297.6±31.2	18.3±0.6	47.0±1.1
Pharbitis purpurea	5:5	**782.3±62.5**	296.0±15.6	16.3±1.2	48.2±8.4
	5:8	1528.5±102.1*	302. 8±36.1	14.9±0.3*	43.3±0.8
Amphicarpaea edgeworthii	5:5	782.0±22.5	279.5±5.6	17.5±0.9	46.5±1.3
	5:8	1238.0±48.8*	258.6±36.1	19.2±0.4*	46.9±1.6

Sorghum halepense	5:5	**781.0±8.6**	283.2±26.5	17.6±0.8	43.9±0.9
	5:8	1129.7±39.5*	287.3±23.8	18.8±1.2	47.7±1.6
Lolium perenne	5:5	**776.9±64.9**	278.7±30.7	19.4±1.2	53.1±3.5*
	5:8	1051.1±113.9*	297.8±14.1	19.8±0.9*	51.1±1.2*
Senecio scandens	5:5	776.2±112.9	291.5±17.9	16.3±0.9	47.1±0.8
	5:8	996.3±53.1*	311.4±25.7	17.2±1.1	45.8±1.6
Cichorium intybus	5:5	759.5±5.5	303.1±9.7	17.4±1.6	52.0±3.4*
	5:8	1154.4±67.1*	288.0±13.7	21. 7±0.5*	50.8±0.8*
Trifolium repens	5:5	758.3±13.7	307.8±11.9	16.4±1.3	47.7±2.1
	5:8	1134.2±69.4*	272.6±8.8	19.5±0.6*	47.4±3.1
Ricinus communis	5:5	751.2±70.0	264.8±18.2	16.0±1.0	44.5±3.2
	5:8	1063.5±62.9*	265.8±19.1	18.5±1.1	46.0±2.6
Euphorbia helioscopia	5:5	750.2±60.1	324.7±11.9	17.5±0.9	47.1±1.2
	5:8	984.4±39.5*	322.8±8.7	16.9±0.7	49.4±1.0
Celosia cristata	5:5	748.7±70. 7	280.0±19.6	15.9±1.2	43.7±5.2
	5:8	1115.3±54.9*	303.9±14.3	20.3±0.5*	51.0±0.4*
Amaranthus retroflexus	5:5	**745.1±127.1**	312.0±26.5	16.2±1.4	47.7±2.7
	5:8	1079.2±113.6*	265.4±12.9	16.3±0.6	46.9±3.1
Aegilops tauschii	5:5	**729.8±30.1**	285.8±5.7	18.1±0.9	47.3±0.8
	5:8	1195.6±103.9*	255.0±24.7	17.9±1.2	46.6±0.7
Cyperus iria	5:5	721.9±90.2	296.5±26.8	16.8±0.8	44.8±1.1
	5:8	1562.8±171.2*	319.2±21.1	23.1±2.0*	52.5±1.2
Conyza bonariensis	5:5	956.3±145.5	277.9±14.5	15.8±0.7	46.0±1.6
	5:8	775.9±57.0	289.2±18.7	15.7±0.6	44.0±2.2
Vicia faba	5:5	952.6±142.3	314.7±7.7	17.8±1.2	46.5±0.9
	5:8	849.5±40.3	278.4±16.9	18.9±0.9	46.3±2.2
Capsella bursa-pastoris	5:5	**950.2±104.7**	334.0±27.2	17.0±2.4	48.9±1.5
	5:8	956.7±55.4	288.3±4.4	18.2±0.8	49.4±0.6
Sonchus arvensis	5:5	**920.2±77.9**	288.4±13.3	17.3±0.6	46.9±1.0
	5:8	906.7±49.1	319.7±20.9	19.2±0.2*	51.3±1.2*
Melilotus officinalis	5:5	917.8±67.1	278.8±20.8	17.9±1.4	48.0±3.0
	5:8	982.8±88.5	301.6±6.9	13.1±0.1*	36.8±1.1*
Bidens pilosa	5:5	883.6±139.8	330.9±47.6	17.1±0.3	44.8±0.5
	5:8	877.8±58.1	306.3±31.4	19.5±0.6*	50.9±1.2*
Commelina communis	5:5	883.4±107.6	282.6±16.1	17.8±1.5	46.9±3.4
	5:8	854.4±54.7	323.6±9.6	16.3±0.6	47.4±0.2
Setaria viridis	5:5	**872.9±131.3**	341.6±21.9	17.4±1.2	45.0±1.6
	5:8	891.7±55.2	314.3±25.3	14.4±0.9*	40.3±2.6
Brassica napus	5:5	853.9±38.7	311.9±49.4	16.3±0.2	50.1±5. 8
	5:8	731.9±108.4	295.8±15.6	18.9±0.3	45.9±0.9
Capsicum annuum	5:5	842.9±60.2	344.8±7.7	17.5±1.1	44.9±3.8
	5:8	957.7±78.8	261.0±50.8	16.5±0.9	45.1±1.6
Abelmoschus esculentus	5:5	839.9±33.9	299.5±5.5	16.8±1.5	47.1±2.2
	5:8	775.1±82.5	289.7±20.7	18.9±0.7	48.2±0.4
Arabidopsis thaliana	5:5	833.9±50.0	268.3±7.2	17.9±0.9	46.2±1.4
	5:8	641.9±95.0	330.2±6.9	19.5±1.9	48.9±2.0

Xanthium sibiricum	5:5	831.8±34.1	336.4±22.3	16.6±1.2	41.7±3.8
	5:8	727.5±62.2	295.6±4.7	20.3±1.1*	47.5±1.1
Celosia argentea	5:5	822.4±28.3	291.9±13.5	16.7±0.4	47.7±2.0
	5:8	969.6±94.0	261.8±12.9	19.6±0.2*	47.2±0.7
Pharbitis nil	5:5	**815.4±74.4**	308.7±3.6	18.1±0.3	49.8±1.3
	5:8	870.8±56.9	221.9±8.9	16.1±0.7	45.3±0.6
Coix lacryma-jobi	5:5	805.4±7.2	298.3±11.0	16.6±1.0	48.3±0. 6
	5:8	795.9±66.9	294.0±14.0	18.0±0.4	48.1±0.9
Sorghum bicolor	5:5	804.4±17.2	297.7±34.8	14.4±1.1*	38.9±3.8*
	5:8	787.0±57.8	257.1±7.6	16.8±0.5	45.8±0.7
Zea mays	5:5	793.2±18.3	282.5±55.4	15.5±0.4	43.8±1.2
	5:8	909.4±67.4	269.6±10.4	16.1±0.5	40.6±1.5
Belamcanda chinensis	5:5	791.5±9.2	279.4±0.6	17.4±0.3	48.2±2.2
	5:8	589.8±4.2	297.1±13.5	21.1±1.1*	52.1±0.8*
Oryza sativa	5:5	790.9±59.5	250.8±15.5	16.4±0.9	47.2±2.6
	5:8	707.9±20.3	286.2±27.5	17.5±1.3	46.2±1.0
Hordeum vulgare	5:5	779.9±63.4	284.6±19.5	16. 9±0.3	45.4±4.8
	5:8	886.3±84.3	302.6±23.5	17.7±1.0	45.6±1.1
Taraxacum mongolicum	5:5	**776.3±96.3**	286.2±20.2	18.6±0.5	46.4±0.4
	5:8	753.5±105.6	301.1±38.5	12.8±0.5*	37.6±0.6*
Cynanchum wilfordii	5:5	773.0±42.4	279.5±21.0	16.9±0.7	48.8±1.4
	5:8	806.6±40.5	282.4±10.5	18.2±0.7	45.4±1.6
Amaranthus mangostanus	5:5	742.8±47.1	320.3±9.3	19.0±1.2	45.9±1.2
	5:8	915.5±86.5	253.0±35.3	18.3±0.7	47.8±1.0
Flaveria bidentis	5:5	729.8±68.9	339.6±5.0	18.5±0.3	45.1±0.2
	5:8	765.5±108.3	320.9±63.6	19.9±0.9*	47.2±3.6
Leptochloa chinensis	5:5	706.5±15.8	301.8±10.2	17.1±0.9	50.1±1.9
	5:8	822.4±101.4	290.2±8.4	20.3±0.8*	49.4±1.7
Rumex japonicus	5:5	**700.4±15.4**	317.9±12.1	17.7±1.2	48.1±1.9
	5:8	896.1±82. 9	319.7±25.3	18.6±0.4	50.0±1.1
Oenothera biennis	5:5	697.3±111.7	317.5±3.7	17.3±0.2	48.9±2.3
	5:8	861.2±87.6	264.6±14.4	18.78±0.3*	48.7±0.4
Solanum nigrum	5:5	**658.2±12. 6**	349.3±22.4	18.4±0.7	43.4±1.3
	5:8	854.3±51.1	266.2±14.9	17.5±1.1	44.9±0.9
Cyperus esculentus	5:5	622.0±74.0	302.6±2.8	17.7±0.2	50.8±0.7*
	5:8	873.5±71.0	281.2±1.4	16.9±0.8	42.2±2.3

Values are means (n=3) ± standard errors (SE). The results of Student's *t*-test for the significance of the difference between wheat-neighbor treatments and wheat-wheat controls at the same mixture proportion are represented by * $P<0.05$. The binomial probability of producing such a large number of significant individual tests was $P<0.0001$ (assuming an α of 0.05) for both 5:5 and 5:8 ratios in DIMBOA production. Significant changes only were observed in 5/100 (binomial probability $P=0.56$) species for wheat shoots and 3/100 species for wheat roots at a 5:5 mixture (binomial probability $P=0.88$). Boldface indicates the data from previously published results, i.e. Zhang, S.Z., Li, Y.H., Kong, C.H. & Xu, X.H. Interference of allelopathic wheat with different weeds. *Pest Manag. Sci.* **72**, 172-178 (2016).

Supplementary Table 3 ANOVA Table for Supplementary Fig. 3

**Effect**	**DIMBOA Concentration**		
	df	**F**	***P***
Species	4, 80	8.8	<0.001
Treatment	3, 80	7.2	<0.001
Mixture proportion	1, 80	265.8	<0.001
Species*Treatment	12, 80	1.4	0.188
Species*Mixture proportion	4, 80	6.8	<0.001
Treatment*Mixture proportion	3, 80	25.2	<0.001
Species*Treatment*Mixture proportion	12, 80	1.3	0.255

Supplementary Table 4 Spectroscopic data of four potential signaling components from the root exudates of neighbor species

Signaling components	^{1}H NMR (600 MHz, $CDCl_3$)	^{13}C NMR (150 MHz, $CDCl_3$)
Jasmonic acid (JA)	*δ* 2.37 (3H, *s*, CH_3), 6.42 (1H, d, *J*=5.4 Hz, pyran-H), 7.71(1H, *d*, *J*=5.4 Hz, pyran-H)	*δ*14.3 (C-7), 113.1 (C-6), 143.2 (C-3), 149.1 (C-2), 154.2 (C-4), 173.0 (C-5)
Salicylic acid (SA)	*δ* 7.84 (1H, *dd*, *J*=0.6,1.2Hz, H-6), 7.40 (1H, *dt*, *J*=0.6,1.2Hz, H-4), 6.88 (1H, *d*, *J*=8.4 Hz, H-3), 6.84 (1H, t, J=7.8, 7.2Hz, H-5)	*δ*114.9 (C-1), 117.9 (C-3),119.8 (C-5), 131.6 (C-6), 136.1 (C-4), 163.0 (C-2), 174.2 (C-7)
(-)-Loliolide ($C_{11}H_{16}O_3$, m/z: 197.2281)	*δ*1.25 (3H, *s*, H-9), 1.36 (3H, *s*, H-11), 1.45 (3H, *s*, H-10), 1.50 (*dd*, *J*=14.5, 3.7 Hz, H-2β), 1.74 (*dd*, *J*=13.8, 4.0 Hz, H-4β), 1.98 (*dt*, *J*=14.5, 2.6 Hz, H-2α), 2.35 (1H, *brs*, OH-3), 2.46 (1H, *dt*, *J*=13.8, 2.6 Hz, H-4α), 4.30 (1H, *m*, *J*=3.3 Hz, H-3), 5.66 (1H, *s*, H-7)	*δ* 26.4 (C-10), 26.9 (C-11), 30.6 (C-9), 36.0 (C-1), 45.6 (C-4), 47.2 (C-2), 66.6 (C-3), 87.1 (C-5), 112.7 (C-7), 172.3 (C-8), 183.1 (C-6)
Luteolin ($C_{15}H_{10}O_6$, m/z: 287.2163)	*δ* 7.39 (1H, *dd*, *J* = 9 Hz, *J* = 2 Hz, H-6′), 7.36 (1H, *d*, *J* = 2 Hz, H-2′), 6.91 (1H, *d*, *J* = 9 Hz, H-5′), 6.54 (1H, *s*, H-3), 6.45 (1H, *d*, *J* = 2 Hz, H-8), 6.21 (1H, *d*, *J* = 2 Hz, H-6)	*δ* 93.5 (C-8), 98.6 (C-6), 103.8 (C-10), 112.6 (C-2′), 115.3 (C-5′), 118.8 (C-6′), 122.6 (C-1′), 145.5 (C-3′), 149.5 (C-4′), 157.9 (C-9), 161.7 (C-5), 164.6 (C-7), 164.8 (C-2), 182.3 (C-4)

High-resolution mass spectrometry (ESI, m/z: $[M+H]^+$) experiments were carried out with IonSpec Ultima FTMS and FABMS instruments with a VG-ZAB-HS (VG Instrument Co., Crawley, UK). The NMR spectra were measured with a Bruker ARX-600 NMR spectrometer (Bruker Instrument Co., Karlsruhe, Germany), and all chemical shifts were reported as δ values relative to the peak for TMS.

Supplementary Table 5 Occurrence of four potential signaling chemicals in wheat and other 100 plant species (Neighbor species consistently arranged in the order of Supplementary Table 2)

	**Potential signaling chemicals (nmol g^{-1} dry weight)**			
**Plant species**	**Jasmonic acid**	**Salicylic acid**	**(-)- Loliolide**	**Luteolin**
Triticum aestivum	2.2±0.2	8.2±0.2	13.1±1.1	37.1±1.8
Raphanus sativus	0.1±0.0	30.9±2.4	1. 9±0.1	1.2±0.2
Glycine max	3.1±0.9	133.0±7.9	5.5±1.0	12.3±1.2
Kummerowia striata	0.4±0.0	24.2±2.5	121.4±8.3	13.5±1.5
Abutilon theophrasti	1.1±0.1	5.5±0.3	9.8±0.3	1.8±0.1
Plantago asiatica	0.2±0.0	1.3±0.1	31.3±1.5	13.7±2.2
Medicago sativa	0.1±0.0	0.2±0.0	4.0±0.9	0.2±0.0
Vigna radiata	0.4±0.1	4.4±0.9	18.6±1.4	0.9±0.1
Kochia scoparia	1.1±0.1	3.4±0.9	56.3±9.9	5. 7±1.1
Daucus carota	0.1±0.0	8.7±1.1	17.2±2.5	0.3±0.1
Orychophragmus violaceus	0.1±0.0	21.7±3.8	31.6±3.9	6.9±1.2
Polypogon fugax	0.4±0.1	3.8±1.0	3.8±0.9	0.1±0.0
Digitaria sanguinalis	5.0±0.3	110.8±8.7	49.1±4.7	27.1±2.0
Cucumis sativus	0.1±0.0	2.2±0.1	86.5±5.5	0.2±0.0
Eleusine indica	4.8±0.7	96.2±5.5	34.4±3.9	ND
Fagopyrum tataricum	0.1±0.0	0.2±0.0	11.0±1.1	ND
Lepidium apetalum	0.3±0.1	41.4±5.0	18.3±4.0	8.5±1.7
Bidens frondosa	0.1±0.0	3.0±0. 9	4.9±0.3	0.6±0.0
Phytolacca acinosa	0.1±0.0	18.8±2.8	9.7±1.4	7.2±1.3
Vicia sativa	0.1±0.0	0.5±0.0	11.2±2.6	ND
Spinacia oleracea	0.1±0.0	1.5±0.9	6.7±1.3	0.2±0.0
Phragmites australis	0.9±0.1	19.0±3.0	81.5±6.7	116.3±8.6
Chrysanthemum coronarium	0.1±0.0	23.0±1.0	100.9±9.5	ND
Hibiscus trionum	0.1±0.0	10.5±2.1	110.4±7.5	2.5±0.9
Cucurbita moschata	0.1±0.0	5.2±0.9	18.8±2.3	0.3±0.0
Descurainia sophia	0.1±0.0	4.2±0.9	10.5±2.3	3.2±1.0
Vigna unguiculata	0.1±0.0	8.0±1.1	4.0±1.0	0.2±0.0
Stipa krylovii	0.2±0.0	48.8±5.3	11.5±2.1	12.0±1.2
Aeschynomene indica	0.1±0.0	24.9±1.3	3.4±0.9	0.9±0.1
Rumex patientia	0.1±0.0	4.9±1.3	17.1±2.0	28.8±2. 8
Chenopodium serotinum	0.1±0.0	8.3±1.7	34.9±4.0	ND
Cirsium setosum	0.09±0.0	9.6±1.3	1.0±0.1	7.3±1.1
Ageratum conyzoides	0.1±0.0	6.2±1.0	1.3±0.8	8.8±1.3
Coriandrum sativum	0.1±0.0	26.6±2.2	28.1±2.2	ND
Chenopodium album	0.1±0.0	6.2±0.9	62.1±4.3	ND
Arctium lappa	0.1±0.0	7.6±1.1	2.1±0.7	0.9±0.1
Cynodon dactylon	0.1±0.0	2.8±0.6	6.1±1.5	0.3±0.1
Lactuca sativa var. ramose	0.1±0.0	3.9±0.9	2.6±0.9	0.7±0.1

Dactyloctenium aegyptium	0.1±0.0	1.2±0.1	20.8±2.6	ND
Echinochloa crusgalli	4.5±0.6	88.2±8.5	21.7±3.2	ND
Avena fatua	6.0±0.3	16.9±1.1	19.0±1.3	42.2±2.8
Senna tora	0.1±0.0	3.0±0.9	16.8±3.5	123.9±9.1
Alopecurus aequalis	0.5±0.1	3.0±0.9	20.8±3.8	ND
Silene conoidea	1.1±0.9	3.6±1.0	51.5±7.4	33.7±5.8
Phaseolus vulgaris	1.0±0.0	2.2±0.9	20.1±3.2	5.0±1.0
Lactuca sativa	0.3±0.1	2.5±1.0	56.5±6.3	7.8±1.9
Artemisia integrifolia	20.9±1.4	9.0±2.3	80.1±4.2	22.4±4.0
Senna occidentalis	0.1±0.0	5.0±1.0	4.6±0.9	0.7±0.1
Alopecurus japonicus	4.5±0.4	127.1±5.2	41.5±1.0	26.7±1.0
Helianthus annuus	0.1±0.0	8.8±1.0	2.6±0.9	0.5±0.1
Lagopsis supina	0.1±0.0	1.3±0.6	8.0±1.9	ND
Gossypium hirsutum	0.1±0.0	1.5±0.7	16.6±2.3	ND
Calystegia hederacea	0.5±0.1	6.2±1.4	15.9±3.5	5.7±1.1
Fagopyrum esculentum	0.1±0.0	1.3±0.9	2.3±0.9	2.1±0.9
Veronica didyma	0.1±0.0	1.8±0.8	27.4±3.0	28.7±2.3
Vaccaria segetalis	0.1±0.0	21.9±4.4	6.0±1.5	1.9±0.9
Eclipta prostrata	0.2±0.1	5.6±0.9	7.9±1.0	2.5±0.9
Pharbitis purpurea	9.3±2.0	4.8±1.0	31.9±8.6	4.1±0.9
Amphicarpaea edgeworthii	1.1±0.1	21.7±3.3	50.9±5.5	4.3±1.1
Sorghum halepense	7.1±1.4	0.8±0.1	67.2±6.5	2.1±1.0
Lolium perenne	3.3±0.1	4.2±0.4	14.2±0.8	3.9±0.2
Senecio scandens	0.9±0.1	0.7±0.1	20.7±2.8	9.1±1.0
Cichorium intybus	0.2±0.1	10.8±1.4	3.1±0.9	3.6±0.9
Trifolium repens	0.1±0.0	1.0±0.1	6.0±1.1	0.2±0.1
Ricinus communis	0.4±0.0	2.3±0.9	2.0±0.8	60.5±5.2
Euphorbia helioscopia	0.2±0.0	6.3±1.0	7.7±1.0	4.2±0.8
Celosia cristata	0.3±0.0	18.4±2.2	29.3±1.3	4.9±0.9
Amaranthus retroflexus	0.5±0.1	10.5±2.4	19.5±2.0	ND
Aegilops tauschii	3.4±0.3	54.1±3.1	109.6±5.6	7.5±0.3
Cyperus iria	1.1±0.1	13.8±1.1	45.6±6.4	6.1±0.3
Chloris virgata	0.1±0.0	1.0±0.1	61.6±7.9	2.1±0.7
Conyza bonariensis	6.6±1.1	4.7±1.0	3.9±0.6	ND
Vicia faba	0.4±0.1	4.8±1.0	22.6±1.2	1.4±0.8
Capsella bursa-pastoris	0.2±0.1	8.0±0.8	19.3±2.2	5.1±0.9
Sonchus arvensis	0.2±0.0	0.4±0.1	80.8±6.5	4.0±1.0
Melilotus officinalis	0.3±0.1	7.2±0.8	20.7±1.9	28.1±1.3
Bidens pilosa	0.1±0.0	0.6±0.0	11.3±1.9	5.9±1.0
Commelina communis	0.1±0.0	5.4±0.9	13.8±1.4	0.4±0.0
Setaria viridis	0.1±0.0	2.0±0.5	21.5±2.3	0.6±0.1
Brassica napus	0.1±0.0	2.1±0.8	6.7±1.0	0.5±0.1
Capsicum annuum	0.3±0.1	2.1±0.9	17.5±5.3	58.5±8.2
Abelmoschus esculentus	0.2±0.0	0.1±0.0	4.5±1.0	0.1±0.0

Arabidopsis thaliana	1.4±0.1	7.0±0.9	70.0±6.9	ND
Xanthium sibiricum	0.1±0.0	0.2±0.0	5.5±1.0	1.2±0.1
Celosia argentea	0.1±0.0	19.7±2.5	54.3±4.4	1.5±0.1
Pharbitis nil	0.2±0.0	0.8±0.5	7.3±1.1	0.4±0.1
Coix lacryma-jobi	0.1±0.0	0.3±0.1	6.9±1.2	0.2±0.1
Sorghum bicolor	0.1±0.0	2.1±0.4	108.3±8.4	1.3±0.4
Zea mays	0.3±0.0	2.9±0.5	7.7±1.0	0.3±0.0
Belamcanda chinensis	0. 1±0.0	5.6±1.0	15.0±2.4	1.6±0.3
Oryza sativa	0.6±0.1	3.7±1.2	1.5±0.4	0.1±0.0
Hordeum vulgare	0.1±0.0	0.2±0.0	10.2±1.6	0.4±0.0
Taraxacum mongolicum	0.2±0.0	19.2±2.3	87.4±6.2	ND
Cynanchum wilfordii	0.1±0.0	9.9±2.4	50.9±8.9	1.2±0.5
Amaranthus mangostanus	0.1±0.0	0.3±0.0	17.2±1.8	ND
Flaveria bidentis	0.3±0.0	28.1±2.3	35.8±6.9	ND
Leptochloa chinensis	0.2±0.0	2.4±0.5	48.8±12.1	7.1±2.4
Rumex japonicus	0.1±0.0	2.4±0.7	33.6±5.0	ND
Oenothera biennis	0.3±0.0	14.1±1.6	67.2±7.8	ND
Solanum nigrum.	0.1±0.0	5.9±0.8	17.9±3.8	ND
Cyperus esculentus	0.6±0.0	6.7±1.2	10.0±1.1	16.3±2.6

ND = not detected. Values (root and shoot combined) are means (n=3) ± standard errors (SE).

Supplementary Table 6 Concentration of potential signaling chemicals in the root exudates and rhizosphere soils (nmol g^{-1} dry soil) for the species used in the exclusion experiment

Plant species	Sources	Potential signaling chemicals (nmol)			
		Jasmonic acid	Salicylic acid	(-)-Loliolide	Luteolin
	Root exudates				
	50 plants 500 mL^{-1}	0.8±0.2	1.9±0.8	0.5±0.2	2.5±0.9
Triticum aestivum	100 plants 500 mL^{-1}	0.8±0.1	2.8±1.2	0.7±0.2	2.8±0.5
	200 plants 500 mL^{-1}	0.9±0.3	3.1±1.4	2.1±0.8	4.4±1.2
	Rhizosphere soil	0.3±0.0	1.2±0.7	0.4±0.1	1.3±0.6
	Root exudates				
	50 plants 500 mL^{-1}	0.4±0.1	5.6±1.8	1.7±0.5	ND
Eleusine indica	100 plants 500 mL^{-1}	0.7±0.2	6.9±2.1	2.2±0.8	ND
	200 plants 500 mL^{-1}	0.7±0.2	8.5±2.4	2.4±0.8	ND
	Rhizosphere soil	0.1±0.0	2.2±0.6	0.3±0.0	ND
	Root exudates				
	50 plants 500 mL^{-1}	0.5±0.1	5.2±1.7	0.9±0.2	2.3±0.8
Digitaria sanguinalis	100 plants 500 mL^{-1}	0.5±0.2	7.7±2.2	1.5±0.6	2.5±0.5
	200 plants 500 mL^{-1}	0.8±0.1	8.2±2.4	2.7±1.0	4.6±1.8
	Rhizosphere soil	0.2±0.0	2.1±0.8	0.9±0.3	1.2±0.0
	Root exudates				
	50 plants 500 mL^{-1}	0.4±0.1	2.2±0.8	1.2±0.6	0.1±0.0
Abutilon theophrasti	100 plants 500 mL^{-1}	0.3±0.1	3.6±1.3	1.4±0.6	0.1±0.0
	200 plants 500 mL^{-1}	0.4±0.1	4.2±1.4	2.8±1.2	0.2±0.0
	Rhizosphere soil	0.1±0.0	1.8±0.6	1.2±0.4	0.1±0.0
	Root exudates				
	50 plants 500 mL^{-1}	0.3±0.1	1.5±0.4	1.0±0.3	0.2±0.0
Bidens frondosa	100 plants 500 mL^{-1}	0.3±0.1	2.2±0.8	1.3±0.6	0.5±0.1
	200 plants 500 mL^{-1}	0.4±0.1	2.3±0.6	2.6±1.1	0.8±0.2
	Rhizosphere soil	0.1±0.0	0.5±0.0	0.8±0.2	0.1±0.0
	Root exudates				
	50 plants 500 mL^{-1}	0.3±0.1	1.3±0.5	1.0±0.4	0.1±0.0
Lolium perenne	100 plants 500 mL^{-1}	0.3±0.1	1.5±0.4	1.6±0.6	0.3±0.0
	200 plants 500 mL^{-1}	0.4±0.1	2.6±0.6	2.0±0.9	0.6±0.1
	Rhizosphere soil	0.1±0.0	0.7±0.2	0.2±0.0	0.1±0.0
	Root exudates				
	50 plants 500 mL^{-1}	0.6±0.1	2.8±0.8	1.6±0.7	2.2±0.7
Avena fatua	100 plants 500 mL^{-1}	0.6±0.1	4.0±1.2	1.8±0.8	2.5±0.8
	200 plants 500 mL^{-1}	0.4±0.1	5.2±1.8	2.8±1.1	4.2±1.1
	Rhizosphere soil	0.1±0.0	1.2±0.0	0.4±0.0	1.4±0.5
	Root exudates				
	50 plants 500 mL^{-1}	0.3±0.1	7.2±1.6	1.0±0.6	1.2±0.4
Alopecurus japonicus	100 plants 500 mL^{-1}	0.3±0.1	8.4±2.2	1.2±0.4	2.1±0.9
	200 plants 500 mL^{-1}	0.5±0. 1	9.2±2.6	2.4±0.8	3.6±1.2

	Rhizosphere soil	0.1±0.0	1.6±0.5	0.3±0.0	0.1±0.0
	Root exudates				
	50 plants 500 mL^{-1}	0.3±0.1	3.0±0.8	1.0±0.3	0.2±0.0
Aegilops tauschii	100 plants 500 mL^{-1}	0.7±0.2	3.2±1.0	1.8±0.6	0.2±0.0
	200 plants 500 mL^{-1}	0.5±0.1	5.8±1.5	2.1±1.0	0.4±0.1
	Rhizosphere soil	0.1±0.0	1.3±0. 4	0.6±0.1	0.1±0.0

ND = not detected. Values are means (n=3) ± standard errors (SE).
